# Mitochondrial oxidative phosphorylation is dispensable for survival of CD34^+^ chronic myeloid leukemia stem and progenitor cells

**DOI:** 10.1038/s41419-022-04842-5

**Published:** 2022-04-20

**Authors:** Jin-Song Yan, Meng-Ying Yang, Xue-Hong Zhang, Chen-Hui Luo, Cheng-Kan Du, Yue Jiang, Xuan-Jia Dong, Zhang-Man Wang, Li-Xue Yang, Yi-Dong Li, Li Xia, Ying Lu

**Affiliations:** 1 Institute of Dermatology, Xinhua Hospital, Shanghai Jiao Tong University School of Medicine, Shanghai, China; 2 Department of Hematology, Liaoning Medical Center for Hematopoietic Stem Cell Transplantation, Liaoning Key Laboratory of Hematopoietic Stem Cell Transplantation and Translational Medicine, Dalian Key Laboratory of Hematology, Diamond Bay Institute of Hematology, the Second Hospital of Dalian Medical University, Dalian, China; 3 Center of Genome and Personalized Medicine, Institute of Cancer Stem Cell, Dalian Medical University, Dalian, China; 4 Department of Pathophysiology, Key Laboratory of Cell Differentiation and Apoptosis of the Chinese Ministry of Education, Shanghai Jiao Tong University School of Medicine, Shanghai, China; 5 Institute of International Medical Science and Technology, Sanda University, Shanghai, China; 6 Department of Core Facility of Basic Medical Sciences, Shanghai Jiao Tong University School of Medicine, Shanghai, China

**Keywords:** Chronic myeloid leukaemia, Haematopoietic stem cells

## Abstract

Chronic myeloid leukemia (CML) are initiated and sustained by self-renewing malignant CD34^+^ stem cells. Extensive efforts have been made to reveal the metabolic signature of the leukemia stem/progenitor cells in genomic, transcriptomic, and metabolomic studies. However, very little proteomic investigation has been conducted and the mechanism regarding at what level the metabolic program was rewired remains poorly understood. Here, using label-free quantitative proteomic profiling, we compared the signature of CD34^+^ stem/progenitor cells collected from CML individuals with that of healthy donors and observed significant changes in the abundance of enzymes associated with aerobic central carbonate metabolic pathways. Specifically, CML stem/progenitor cells expressed increased tricarboxylic acid cycle (TCA) with decreased glycolytic proteins, accompanying by increased oxidative phosphorylation (OXPHOS) and decreased glycolysis activity. Administration of the well-known OXPHOS inhibitor metformin eradicated CML stem/progenitor cells and re-sensitized CD34^+^ CML cells to imatinib in vitro and in patient-derived tumor xenograft murine model. However, different from normal CD34^+^ cells, the abundance and activity of OXPHOS protein were both unexpectedly elevated with endoplasmic reticulum stress induced by metformin in CML CD34^+^ cells. The four major aberrantly expressed protein sets, in contrast, were downregulated by metformin in CML CD34^+^ cells. These data challenged the dependency of OXPHOS for CML CD34^+^ cell survival and underlined the novel mechanism of metformin. More importantly, it suggested a strong rationale for the use of tyrosine kinase inhibitors in combination with metformin in treating CML.

## Introduction

Chronic myeloid leukemia (CML) are hematopoietic malignancies that arise from transformation of hematopoietic stem cells (HSC) driven by chimeric oncoprotein BCR-ABL, a constitutively active tyrosine kinase generating from the t(9;22)(q34;q11) chromosomal translocation [1–3]. Applications of tyrosine kinase inhibitors (TKIs) including imatinib, nilotinib, and dasatinib have dramatically improved the life expectancy of CML patients [4]. However, these drugs do not kill the leukemia stem cells (LSC) that maintain CML [5–9], resulting in resistance and disease relapse, highlighting the urgent need to overcome the insensitivity of leukemic stem/progenitor cells to TKI for treating this disease.

As a hallmark of cancer stem cells, metabolic reprograming has been an actively investigated target for therapeutic purpose. However, compared to extensive studies on acute myeloid leukemia (AML) stem/progenitor cells, the metabolic signatures of CML stem/progenitor cells are limited. Stable isotope-assisted metabolomics demonstrated oxidative metabolism dependency for the survival of primitive CML cells [10]. In contrast, dependency of CML stem/progenitor cells on aerobic glycolysis was observed in MLL-AF9 and BCR-ABL transformed murine leukemia models [11]. More recently, aberrantly activated branched-chain amino acid aminotransferase 1 (BCAT1) and branched-chain amino acids (BCAAs) metabolism are reported to be functionally required for progression of CML in both human and mouse models [12]. On the other hand, extensive studies focusing on the genetic and transcriptomic mechanism responsible for metabolic aberrations of leukemia stem/progenitor cells have been conducted [13–19]. However, increasing evidence showed poor correspondence between mRNA and proteins in multiple tumors. The Clinical Proteomic Tumor Analysis Consortium (CPTAC) work also found inconsistent correspondence between mRNA and protein abundance, emphasizing that to determine mRNA levels of metabolic enzymes is insufficient for a complete understanding of the cancer stem and progenitor cells [20–26]. Herein, to map changes in protein levels associated with metabolic signature, we integrated label-free quantitative proteomic analysis, transcriptomic data, and metabolic activity assays, aiming to search for CD34^+^ leukemia cell-specific metabolic vulnerabilities for therapeutic purpose.

## Materials and methods

### Patients and cells

Bone marrow samples were collected from 11 cases of newly diagnosed CML patients at the Department of Hematology of the Second Hospital of Dalian Medical University. Patients were diagnosed according to French–American–British classification. Informed consent was obtained from all patients in accordance with the Declaration of Helsinki, and all manipulations were approved by the Medical Science Ethic Committee of Dalian Medical University. Mononuclear cells were isolated by density gradient centrifugation using Lymphoprep, and cryopreserved. In addition, 5 potential donors for allogeneic bone marrow transplantation were used to purify healthy hematopoietic cells. Human CD34^+^ cells were enriched from bone marrow mononuclear cells using MiniMACS (Miltenyi Biotech, Bergisch Gladbach, Germany) following the manufacturer’s instructions [27]. Confirmation of CD34^+^ cells phenotype and purity was assessed by flow cytometry analysis using CD34-PE-Cy7 (BD Biosciences, San Diego, CA). Purified CD34^+^ cells were grown in serum-free hematopoietic growth medium (HPGM; Lonza) supplemented with 10 ng/mL recombinant human interleukin 3 (rhIL-3), 10 ng/mL rhIL-6, and 50 ng/mL recombinant human stem cell factor (PeproTech) in a humidified incubator at 37 °C and 5% CO_2_/95% air (v/v).

### Reagents and antibodies

Imatinib (HY-15463) and metformin (HY-B0627) and tunicamycin (HY-A0098), MKC8866 (HY-104040) were obtained from MedChemExpress (MCE). Annexin V/PI staining kit was purchased from Thermo Fisher Scientific (BMS500FI-100). Antibodies against the following proteins were used: CD34 (Abcam, ab81289), IRE1α (endoribonuclease α, Proteintech, 27528-1-AP), PERK (Protein kinase R-like endoplasmic reticulum kinase, Proteintech, 24390-1-AP), Phospho-PERK (p-PERK, Thr982, Beyotime Biotechnology, AF5902), and β-actin (Cell Signaling Technology, #4970).

### Real-time quantitative RT-PCR

Total cellular RNA from Primary CD34^+^ cells of healthy donors and CML patients we extracted by TRIzol reagent (ER501-01, TransGen Biotech). Complementary DNA (cDNA) was synthesized using the cDNA synthesis kit according to the manufacturer’s instructions (AE341-02, TransGen Biotech). Quantitative real-time PCR was performed using SYBR Green PCR master mix (11202ES08, YEASEN) on a QuantStudio 6 real-time PCR system (Applied Biosystems). Data were analyzed by the 2^−∆∆CT^ method and were normalized to the expression of the control gene *GAPDH*. Specific oligonucleotide primers for *XBP1s* (spliced X-box binding protein 1), *ERDJ4* (endoplasmic reticulum DNA J domain-containing protein 4), *ATF6* (activating transcription factor 6), *CHOP* (C/EBP-homologous protein), *GADD34* (growth arrest and DNA-damage-inducible 34), *HSP90B1* (heat shock protein 90 beta family member 1), *BIP* (binding-immunoglobulin protein) were used and listed below. The *XBP1s* primer sequences were from the literature [27], and the rest of the primers were designed using NCBI (National Center for Biotechnology Information) Primer-BLAST. *GAPDH* cDNA was amplified as an internal control (forward primer: 5’-TGCACCACCAACTGCTTAG-3’; reverse primer: 5’-GGATGCAGGGATGATGTTC-3’)

*XBP1s*-F: 5’-AGTCCGCAGCAGGTGCAG-3’,

*XBP1s*-R: 5’-CTTCCAGCTTGGCTGATGAC-3’;

*BIP*-F: 5’-AAGCCCGTCCAGAAAGTGTT-3’,

*BIP*-R: 5’-GACAGCAGCACCATACGCTA-3’;

*HSP90B1*-F: 5’-GGATGGTCTGGCAACATGGA-3’,

*HSP90B1*-R: 5’-CCGAAGCGTTGCTGTTTCAA-3’;

*ERDJ4*-F: 5’-CAGAGCGCCAAATCAAGAAGG-3’,

*ERDJ4*-R: 5’-CTTCAGCATCCGGGCTCTTAT-3’;

*ATF-6-*F: 5’-CTGTTACCAGCTACCACCCA-3’,

*ATF-6*-R: 5’-GGGGAGCCAAAGAAGGTGTT-3’;

*CHOP*-F: 5’-TGTTCCAGCCACTCCCCATTAT-3’,

*CHOP*-R: 5’-GTGTCCCGAAGGAGAAAGGC-3’;

*GADD34*-F: 5’-CTGGCTGGTGGAAGCAGTAA-3’,

*GADD34*-R: 5’-TATGGGGGATTGCCAGAGGA-3’;

### Short-term colony-forming cell (CFC) assay

Purified primary CD34^+^ cells were cultured in Stemspan serum-free medium (Stem Cell Technologies) containing 10 ng/mL human SCF, and 10 ng/mL human TPO (Peprotech) in the presence of metformin. Forty-eight hours after the culture, the cells were transferred to a methylcellulose-based medium (Methocult H4034 Optimum, StemCell Technologies) in triplicate, and colonies were manually counted after 10–14 days.

### Cell counting and apoptosis assays

Purified primary CD34^+^ cells were seeded at 1 × 10^6^ cells/ml before drug treatment and counted by trypan blue (Sigma-Aldrich) exclusion. Apoptosis was quantified by staining with Annexin V-FITC and PI (BD Biosciences, 556547).

### Mass spectrometry (MS) analysis

The eluted peptides were lyophilized using a SpeedVac and resuspended in 10 μl 1% formic acid/5% acetonitrile. All mass spectrometric experiments are performed on a Orbitrap Fusion LUMOS mass spectrometer (Thermo Fisher Scientific) connected to an Easy-nLC 1200 via an Easy Spray (Thermo Fisher Scientific). The peptides mixture was loaded onto a self-packed analytical PicoFrit column with an integrated spray tip (New Objective) (75 μm × 20 cm length) packed with 130 A C18 1.7 μm (waters) and separated within a 60 min’ linear gradient from 95% solvent A (0.1% formic acid/2% acetonitrile/98% water) to 28% solvent B (0.1% formic acid/80% acetonitrile) at a flow rate of 300 nl/min at 50 °C. The spray voltage was set to 2.1KV and the temperature of ion transfer capillary was 275 °C, and RF lens was 40%. The mass spectrometer was operated in positive ion mode and employed in the data-dependent acquisition (DDA) mode within the specialized cycle time (3 s) to automatically switch between MS and MS/MS. One full MS scan from 350 to 1500 *m/z* was acquired at high-resolution R = 120,000 (defined at *m/z* = 400); MS/MS scans were performed at a resolution of 30,000 with an isolation window of 4 Da and higher-energy collisional dissociation (HCD) fragmentation with collision energy of 30% ±5. Dynamic exclusion was set to 30 s.

### MS data processing

All MS/MS ion spectra were analyzed using PEAKS X (Bioinformatics Solutions) for processing, de novo sequencing, database searching, and label-free quantification. Resulting sequences were searched against the UniProt Human Proteome database (downloaded 5 May 2018) with mass error tolerances of 10 ppm and 0.02 Da for parent and fragment, respectively, the digestion enzyme semiTrypsin allowed for two missed tryptic cleavages, Carbamidomethyl of cysteine specified as a fixed modification, and Oxidation of methionine, acetyl of the N-terminus and phosphorylation of tyrosine, serine and threonine as variable modifications. FDR estimation was enabled. Peptides were filtered for −10log *P* ≥ 15, and proteins were filtered for −10log *P* ≥ 15 and one unique peptide. For all experiments, this gave an FDR of <1% at the peptide-spectrum match level. Proteins sharing significant peptide evidence were grouped.

### CML patient-derived xenograft models

For the ex vivo drug studies, CML cells (1 × 10^6^ cells per mouse) were transplanted via tail vein into female 8–10-week-old sub-lethally irradiated (2.5 Gy) NOD.Cg-Prkdc^scid^Il2rg^tm1Wjl^/SzJ NSG mice (The Jackson Laboratory). The mice were intraperitoneally injected with 50 mg/kg/day of metformin, within the clinical dose range for 14 days. Imatinib was intraperitoneally injected at 50 mg/kg/day for 14 days. Human cells were assessed using anti-human CD45 (Invitrogen, 11045942), anti-mouse CD45 (Invitrogen, 12045182), anti-human CD34 (Invitrogen, 17034942), anti-human CD33 (Invitrogen, 48033742) by flow cytometry. Animal handling was approved by the committee for humane treatment of animals at Shanghai Jiao Tong University School of Medicine.

### Mitochondrial stress assay

Oxygen consumption rate (OCR) was measured using XF Cell Mito Stress Test Kit (Agilent Technologies). Purified CML cells were seeded in an XF96 cell culture microplate or treated with metformin. The sensor cartridge and base medium were prepared by adding 1 mmol/L pyruvate, 2 mmol/L glutamine, and 10 mmol/L glucose and stored as per the manufacturer’s instructions [28]. Seahorse assay was run in XF96 Extracellular Flux Analyzer (Agilent Technologies). Following three baseline OCR measurements, cells were exposed sequentially to oligomycin (0.5 mmol/L), carbonyl cyanide-4 (trifluoromethoxy) phenylhydrazone (FCCP; 1 mmol/L), and rotenone/antimycin A (0.5 mmol/L). The results were analyzed using Wave program 2.3.0 (Seahorse Bioscience) after being normalized with cell number/well.

### Glycolysis stress assay

The extracellular acidification rate (ECAR) was measured by the Seahorse XF Glycolysis Stress Test Kit (Agilent Technologies). Purified CML cells were seeded in a XF96 cell culture microplate or treated with metformin. The sensor cartridge and assay medium preparation were performed as per the manufacturer’s instructions. Following three baseline ECAR measurements, cells were exposed sequentially to glucose (10 mmol/L), oligomycin (1.0 mmol/L), and 2-deoxy-glucose (50 mmol/L). Three measurements were recorded after every injection. The results were analyzed using Wave program 2.3.0 (Seahorse Bioscience) and were normalized with cell number/well.

### Statistical analysis

Statistical analyses between the control and treatment groups were performed by standard two-tailed Student’s *t* test. All experiments were repeated at least three times. A value of *P* < 0.05 was considered to be statistically significant.

## Results

### Buildup of metabolic proteome of CML stem/progenitor cells

**Fig. 1:**
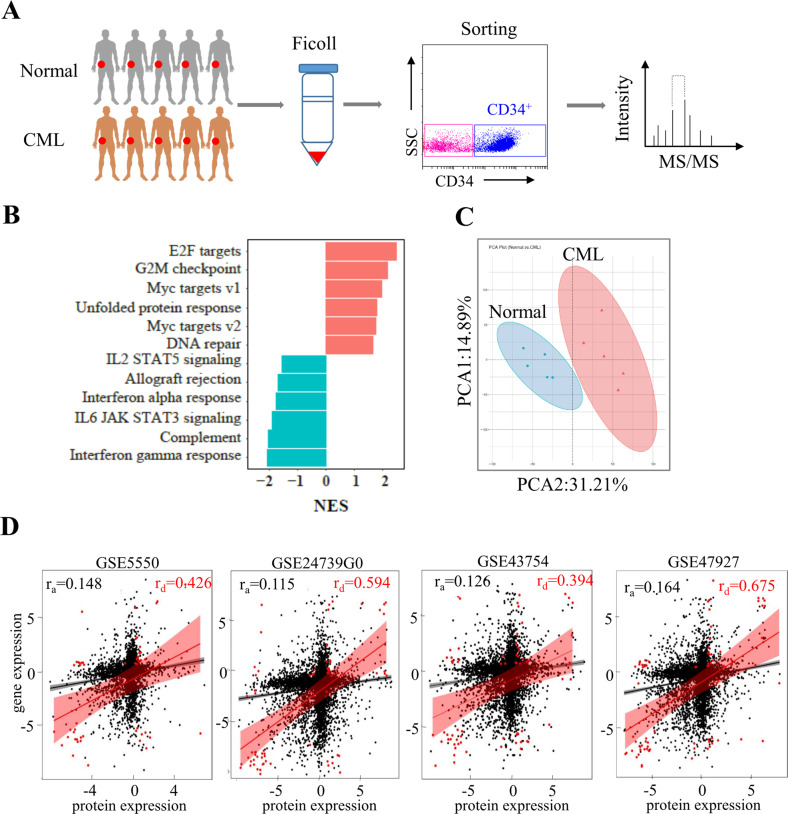
Metabolic proteome signature of CD34^+^ leukemia cells. **A** Diagram of experimental design. **B** Bar plots of top-ranked HALLMARK pathways that are significantly altered in CD34^+^ leukemia samples compared with the normal counterpart. NES: Normalized Enrichment Score. **C** The 2D plot of PCA between the CD34^+^ CML samples and normal samples. The differentially expressed proteins were used to carry out the analysis under the threshold of FC (fold change) >2 and *P* < 0.01. PCA1 and PCA2 denote the first and second principal component, respectively. **D** Correlation coefficient was calculated between the CD34^+^ cells protein log2 ratios (*n* = 5 patient samples, *n* = 5 normal samples) and transcript logFC in the four datasets (GSE24739, GSE43754, GSE47927, and GSE5550) using Pearson method. Filled black circles indicate all proteins/genes; filled red circles indicate regulated proteins/genes (FC > 2, *P* < 0.001). r_a_ all proteins/genes correlation, r_d_ deregulated proteins/genes correlation.

Several reports described the metabolome of leukemia stem cells but none have clarified the transcriptional or post-transcriptional origin of metabolic signature [29–31]. To elucidate the metabolic proteome that underlies CML progression, we purified CD34^+^ stem/progenitor cells from treatment-naive CML as well as normal CD34^+^ cells and performed label-free quantitative proteomic analysis (Fig. 1A). Gene set enrichment analysis (GSEA) based on the protein abundance found that CD34^+^ CML cells were predominantly enriched for E2F targets, G2M checkpoint, MYC targets pathways, unfolded protein response and DNA repair (Fig. 1B). Given the clear important role of these subsets of protein in controlling cell cycle transitions, it strongly suggested that aberrant cell growth is the driver for CML CD34^+^ cell transformation. Next, we obtained the differentially expressed proteins by comparing the CML CD34^+^ with normal CD34^+^ samples (Supplemental Fig. 1). The principal component analysis (PCA) showed robust segregation between CML CD34^+^ from normal CD34^+^ proteomes (Fig. 1C). To further investigate regulation in leukemia stem/progenitor cells, we examined correlation between proteome with primary CML LSC transcriptomic datasets. Whole protein levels did not show significant correlation with respective gene levels (four independent datasets for CML) in CML CD34^+^ progenitors (Fig. 1D). In contrast, all the significantly deregulated proteins (fold change >2) correlated well with respective gene counterpart (Fig. 1D), suggesting a transcriptional mechanism for deregulated proteins in its entirety.

**Fig. 2:**
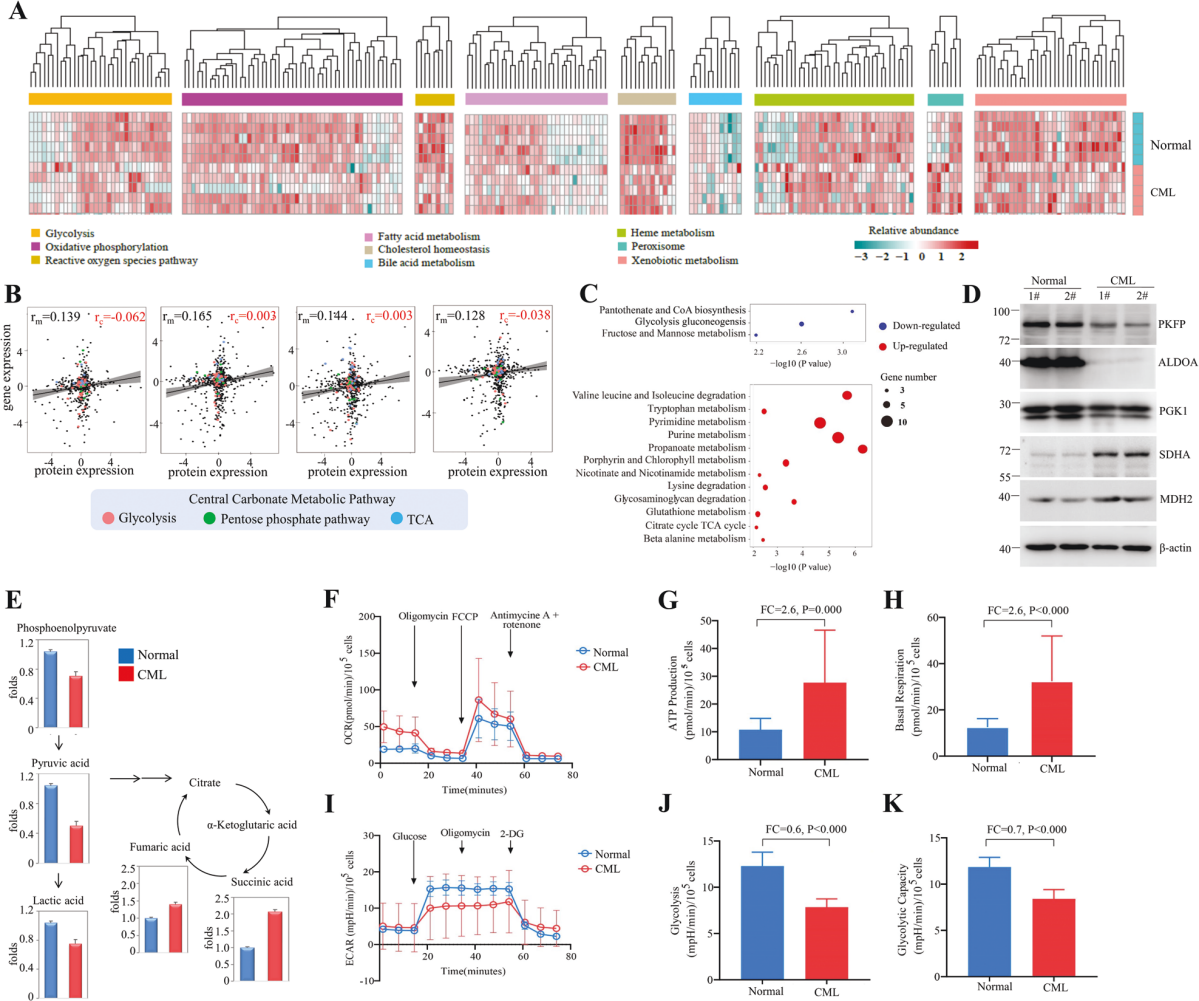
CD34^+^ CML cells showed increased OXPHOS and decreased glycolysis. **A** Hierarchical clustering analysis for nine HALLMARK metabolic pathways between CD34^+^ CML and normal samples. Clustering was done using Euclidean distance and complete linkage method with the differentially expressed genes (FC > 2 and *P* < 0.01) that annotated in the metabolic pathways. Quantile normalization was applied prior to the analysis. **B** Correlation between metabolic proteomic/transcriptomic in primitive CML CD34^+^ cells using Pearson method. Filled black circles indicate all metabolic proteins/genes in KEGG (*n* = 1,480); filled red, green, and blue circles indicate proteins/genes in central carbonate metabolic pathway (*n* = 101). r_m_ all metabolic proteins/genes correlation, r_c_ central metabolic proteins/genes correlation. **C** Bubble plot representation of selected KEGG pathways that are enriched in the metabolic categories for CD34^+^ CML samples. The size of each bubble corresponds to the number of genes within the given gene set. The pathways enriched in upregulated genes are colored in red, and pathways enriched in downregulated genes are colored in blue. **D** Western blot analysis of proteins in TCA and glycolysis. **E** Comparative metabolomics analyses of primary CD34^+^ purified from CML or normal donors performed via LC-MS. Data are shown as the mean from *n* = 3 experiments. **F** Oxygen consumption rate (OCR) levels were examined in normal or CML CD34^+^ using a Seahorse XF96 analyzer (*n* = 3). **G**, **H** ATP levels and basal respiration were examined. **I** Extracellular acidification rates (ECAR) of CD34^+^ purified from CML or normal donors were measured by Seahorse Glycolysis Stress Kit. **J**, **K** Level of glycolysis and glycolytic capacity were examined.

Next, we dissected the metabolic proteome of CML CD34^+^ stem/progenitor cells. The heatmap of nine HALLMARK metabolic pathways in the Molecular Signatures Database (MSigDB) showed aberrant expression of glycolysis, oxidative phosphorylation, cholesterol homeostasis, bile acid and heme metabolic pathways in CML CD34^+^ cells (Fig. 2A). Interestingly, no correlation was observed between metabolic proteomic and transcriptomic data, suggesting that metabolic proteins were not regulated at the transcriptional level (Fig. 2B). KEGG pathway analysis revealed that CML stem/progenitor cells have downregulated glycolysis proteins and upregulated TCA proteins (Fig. 2C), supporting previous research showing that CML stem cells rely on upregulated oxidative metabolism for their survival yet with undefined mechanism [32]. Further Ingenuity Pathway Analysis (IPA) analysis focusing on central carbonate metabolic pathways demonstrated that multiple proteins in TCA cycle [SUCLG1 (succinyl-CoA ligase [ADP/GDP-forming] subunit alpha, mitochondrial), SDHA (succinate dehydrogenase A), MDH2 (malate dehydrogenase 2)] are upregulated, accompanying by downregulation of glycolysis enzymes [PKFP (phosphofructokinase), ALDOA (fructose-bisphosphate aldolase A), GAPDH (glyceraldehyde 3-phosphate dehydrogenase), PGK1 (phosphoglycerate kinase 1), PGAM1 (Phosphoglycerate Mutase 1)] and upregulation of rate-limiting enzyme glucose-6-phosphate dehydrogenase (G6PD) in phosphate pentose pathway (PPP) (Supplemental Fig. 2). These results were recapitulated by western blot analysis (Fig. 2D) and measurement of metabolites central to glucose metabolism through liquid chromatography (LC)-MS showing that the abundance of metabolites from the glycolysis pathway including phosphoenolpyruvate (PEP), pyruvic acid, and lactic acid were decreased accompanying by an increase of TCA metabolites, including fumaric acid and succinic acid (Fig. 2E).

Moreover, seahorse analysis showed that instead of displaying the Warburg effect as most solid tumor cells do, the CML CD34^+^ cells had higher oxygen consumption rates (OCRs), mitochondrial membrane potential, and ATP levels (Fig. 2F–H) and lower level of ECAR, glycolysis rate, and glycolytic capacity (Fig. 2I–K) than normal CD34^+^ hematopoietic cells. Together, these data suggested that the metabolic signature of CML CD34^+^ cells may have originated from protein level and re-balancing glucose metabolic pathways may have a therapeutic benefit.

### Metformin kills CML CD34^+^ cells

**Fig. 3:**
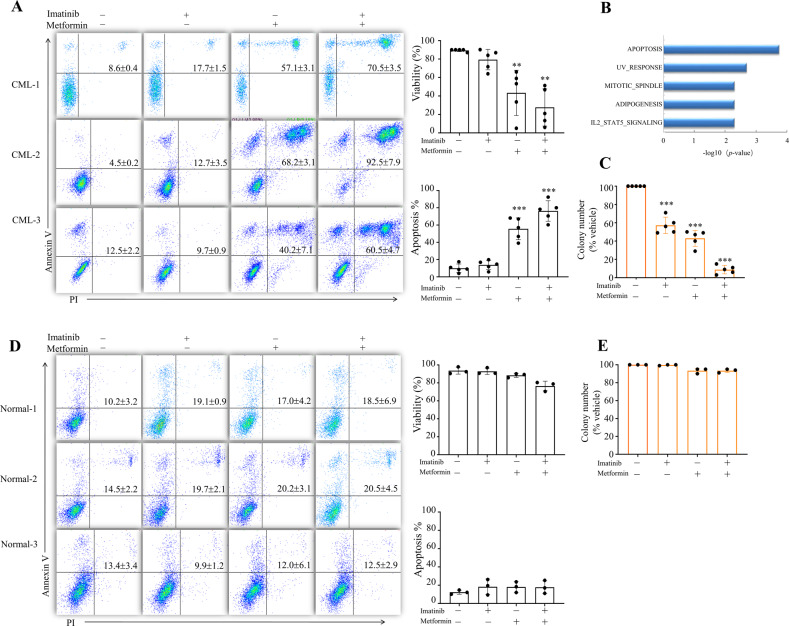
Metformin induces apoptosis of cultured CD34^+^ CML cells. **A** Purified CD34^+^ cells from CML patients were treated with metformin, imatinib or metformin plus imatinib for 72 h. Cell apoptosis were measured by Annexin V/PI staining. Representative flow cytometry plots of CD34^+^ cells from three CML individuals were shown. Cell viability measured by trypan blue (*n* = 5 patient samples) and quantified apoptosis (*n* = 5 patient samples) were shown on the right. **B** Purified CD34^+^ cells from CML patients were treated with metformin for 24 h and subjected to label-free quantitative MS analysis. Top five significant enrichment pathways after metformin treatment are shown. The differentially expressed proteins were used to carry out the analysis under the threshold of FC > 1.5 and *P* < 0.05. **C** CFCs of CD34^+^ CML cells from patients treated with metformin, imatinib or metformin plus imatinib for 48 h. Colony numbers were shown. *n* = 5 patient samples. ** and *** indicated *P* value against 0 or control group <0.005 and <0.001, respectively. **D**, **E** Purified CD34^+^ cells from normal donors were treated with metformin, imatinib or metformin plus imatinib for 72 h. Cell apoptosis (**D**) and CFCs (**E**) were measured. Representative flow cytometry plots are shown on the left (**D**). Cell viability measured by trypan blue and quantified apoptosis (*n* = 3 normal samples) on the right (**D**).

To rewire the metabolic signature of CML, we selected the most prescribed anti-diabetic drug metformin, which is widely recognized to suppress TCA yet through undefined mechanism. Treatment with 2 mΜ metformininduced apoptosis of CD34^+^ cells purified from three CML individuals (Fig. 3A). Consistent with the deleterious effects of metformin, we observed enrichment for protein expression signatures associated with apoptosis (Fig. 3B). More importantly, administration of metformin leads to synergistic kill with imatinib, which failed to eradicate CD34^+^ CML cells (Fig. 3A). In line with this observation, treatment with metformin alone decreased the number of short-term CML colony-forming cell (CFC) and metformin with imatinib in combination effectively eliminated colony formation (Fig. 3C). These data suggested that metformin endows CML stem/progenitors with sensitivity to imatinib. We next evaluated the toxicity of metformin at millimole level on normal CD34^+^ and observed less death and CFC impair of normal CD34^+^ compared to its CML counterpart (Fig. 3D, E), indicating a therapeutic window. In addition, in contrast to primitive CML CD34^+^ cells, treatment of metformin did not induce apoptosis of human CML K562 cell lines but arrested cell cycle at the S phase after 48 h treatment (Supplemental Fig. 3) showing a discrepancy between CML cultured cell lines and primary cells.

### Mechanism of metformin on CD34^+^ CML cells

**Fig. 4:**
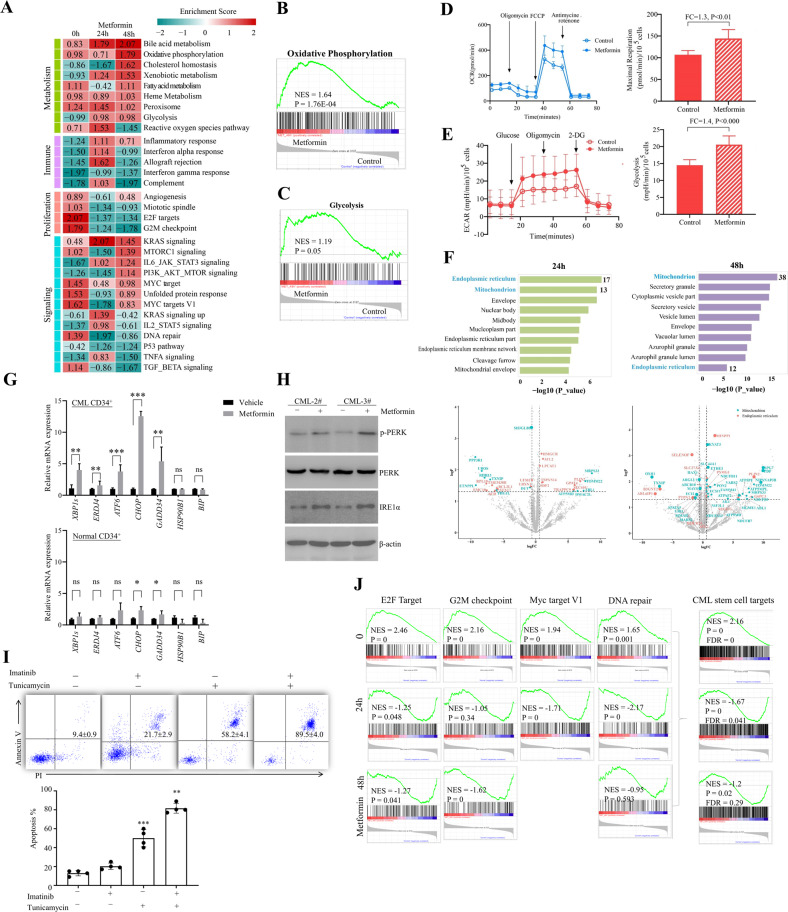
Mechanism of metformin on CML CD34^+^ cells. **A** Purified CML CD34^+^ cells were treated with metformin for 24 and 48 h and subjected to label-free quantitative MS analysis. Heatmap of alternation pathways regulated by metformin was shown. **B**, **C** Representative GSEA plots of oxidative pathway and glycolysis post metformin treatment 48 h. **D**, **E** OCR and ECAR of CML CD34^+^ cells in response to metformin. **F** GO cell compartment (CC) enrichment analysis and volcano plots of proteins regulated by metformin for indicated times (FC > 1.5 and *P* < 0.05). **G**, **H** RT-PCR (**G***, n* = 3) and western blot analysis (**H**) of ER stress signaling of metformin or vehicle-treated purified CML or normal CD34^+^ cells. *, **, and *** indicated *P* value< 0.05, <0.005, and <0.001, respectively. **I** Purified CML CD34^+^ cells were treated with imatinib, tunicamycin or tunicamycin plus imatinib for 72 h. Cell apoptosis were measured. Representative flow cytometry plots were shown on the top. *n* = 4. ** and *** indicated *P* value <0.005 and <0.001 versus non-treated group, respectively. **J** GSEA plots of E2F target, G2M checkpoint, MYC target, DNA repair, and combined protein subset named CML stem cell targets are shown.

To understand the mechanism underlying apoptosis-inducing effect of CML stem/progenitor cells in response to metformin, quantitative proteomics were performed using CD34^+^ cells purified from three CML individuals and treated with metformin for 24 and 48 h. Functional pathway enrichment analysis identified multiple subsets of proteins involved in metabolism, immune, proliferation and signaling were modulated by metformin (Fig. 4A). Surprisingly, we observed that metabolic protein signatures associated with central carbonate metabolic pathways of CML CD34^+^ cells, including decreased glycolysis and increased TCA were not fully reversed by metformin. The abundance of both glycolysis and OXPHOS proteins were upregulated upon metformin treatment (Fig. 4B, C). Using the seahorse technique, we measured the OCR and ECAR of CML CD34^+^ cells derived from three individuals treated with metformin. Consistent with the proteomic results, metformin upregulated both ECAR and OCR of CD34^+^ CML cells (Fig. 4D, E). These data suggested that the impact of metformin on CD34^+^ CML cells is not through the well-known mechanism on mitochondria metabolism.

The mechanisms underlying the action of metformin are complicated and still not fully understood. At the molecular level, metformin has been shown to act toward mitochondrial targets including Complex I of the respiratory chain and glycerol-3-phosphate dehydrogenase (GPD2), as well as cytoplasm target AMP-dependent kinase (AMPK) due to modulation of AMP/ATP ratio [33–38]. Interestingly, top enrichment of cell component 24 h post metformin treatment is endoplasmic reticulum (ER) proteins with 17 of which have been proposed to modulate by metformin (Fig. 4F). To validate the activation of ER stress, we treated CML CD34^+^ and normal progenitors with metformin and detected markers of ER stress signaling. We observed a significant increase in the level of p-PERK and IRE1α accompanied by a marked upregulation of the ER chaperons in CML progenitors but not its normal counterparts (Fig. 4G, H) [39]. Co-treatment of MKC8866 could partially block the apoptosis-inducing effect of metformin in purified CML CD34^+^ cells (Supplemental Fig. 4). Furthermore, ER stress inducer tunicamycin presented synergistic effect with imatinib further supporting that ER stress account for, at least partially the effect of metformin (Fig. 4I). Considering that metformin could induce cell death in an ER stress-dependent way and the ER-associated events occurs earlier than that of mitochondrial (Fig. 4F) [40–42], we proposed that the apoptosis-inducing effect is not due to the metabolic rewiring function of metformin in CML stem/progenitor cells. In consistent with our finding, multiple studies reported that metformin-induced ER stress followed by impairment of mitochondrial integrity and function [40–45]. More intriguingly, the top four aberrantly expressed pathways in CML stem/progenitor cells including E2F targets, G2M checkpoint, DNA repair and MYC targets pathways (Fig. 1B) were remarkably downregulated (Fig. 4J). Grouping these four pathways into one subset, referred to as “CML stem cell targets” here, we observed a more significant downregulation by metformin (Fig. 4J), indicating that interfered cell growth drive cell killing of metformin.

### Metformin re-sensitizes CD34^+^ CML cells to imatinib in PDX model

**Fig. 5:**
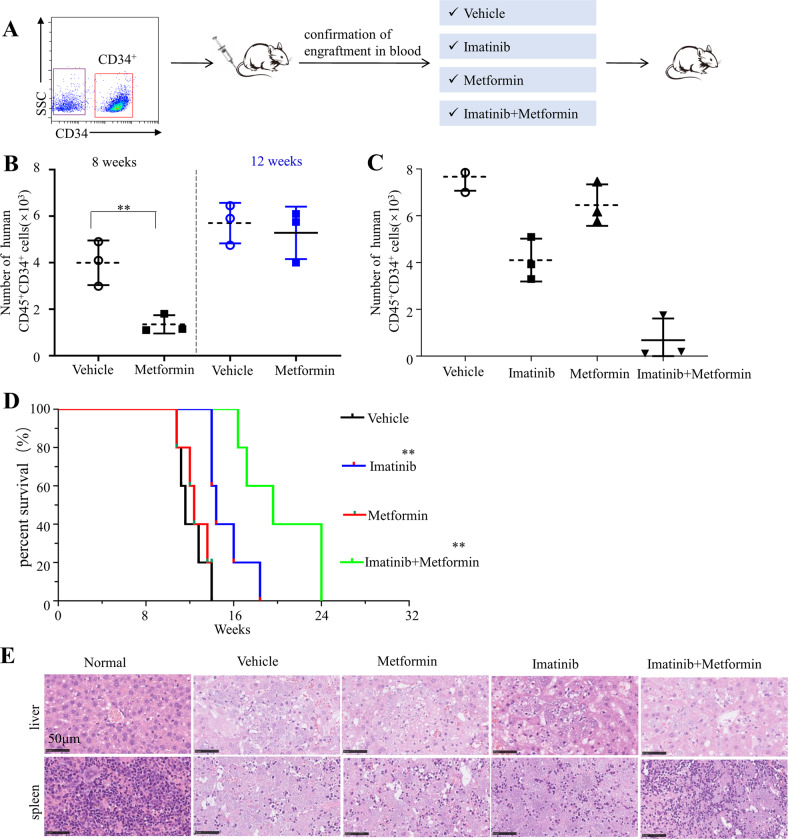
Metformin resumes the sensitivity of CD34^+^ CML cells to imatinib in the PDX model. **A** Diagram of experimental design. The engraftment of CML CD34^+^ cells in mice were assessed by monitoring the percentage of human CD45^+^ circulating leukocytes using flow cytometry. **B**, **C** Percentages of human CD45^+^CD34^+^ cells in the bone marrow post transplantation are shown. **D** Survival of the CML PDX mice with indicated treatment was shown. **E** The leukemic invasions in the spleen and liver of PDX mice with indicated treatment were analyzed by hematoxylin and eosin (H&E) staining.

To assess the clinical relevance of these findings, the in vivo effects of metformin on CD34^+^ leukemia stem/progenitor cells were further evaluated by patient-derived xenograft (PDX) CML models. CD34^+^ cells were purified from BMMC of CML patients and transplanted into sub-lethally irradiated NSG mice (Fig. 5A). Human CD45^+^ cells were detectable by flow cytometry in peripheral blood at 6 weeks post transplantation. The mice were treated with vehicle, metformin (50 mg per kg body weight), imatinib (100 mg per kg body weight) or both compounds for 14 days. No change in random blood glucose level was observed (data not shown). Expression of human CD45 and CD34 were measured by flow cytometry in the bone marrow. Administration of metformin clearly reduced engraftment of CML stem/progenitor cells as indicated by decreased numbers of CD45^+^CD34^+^ cells in the bone marrow of CML PDX models at 8 weeks post transplantation (Fig. 5B). However, the percentage of CD45^+^CD34^+^ cells returned to comparable level with vehicle at 12 weeks post transplantation (Fig. 5B). In contrast, combination of metformin with imatinib eliminated 90% of CD45^+^CD34^+^ population as detected at the end point (the time point that the mice were sacrificed) (Fig. 5C). Correspondingly, the metformin monotherapy failed to significantly prolong the survival of CML CD34^+^ cell transplanted mice, whereas the combination of metformin with imatinib effectively retarded the engraftment of human CD34^+^ stem/progenitor cells, leading to significantly longer survival (Fig. 5D). In support of this observation, histological examination revealed that combination treatment inhibited the infiltration of leukemia cells into the spleen and liver (Fig. 5E). Together, these results suggested that metformin endows CML CD34^+^ cells with sensitivity to imatinib in vivo.

## Discussion

It is well recognized that cancer stem cells present unique metabolic signature compared to bulk population, thus providing vulnerability for targeting therapy to eradicate these roots of cancer. Oxidative phosphorylation, glycolysis, fatty acid oxidation (FAO), amino acid metabolism dependency of leukemia stem cells have all been reported, leading to a number of metabolism inhibitors undergoing preclinical evaluations in treating leukemia. On the other hand, despite extensive genomic, transcriptomic, or metabolic studies, the question with respect to from what level the metabolism was reprogrammed remained unanswered. Complicating matter is that mRNA expression and protein abundance are generally not well correlated across yeast and higher eukaryotes [20, 22, 23]. Data from a most recent proteomic study also demonstrated that mouse HSCs exhibit minimal regulation at the genomic and transcript level [22, 46]. Therefore, a deep understanding of metabolic signature of stem/progenitor cells undoubtedly requires proteomics characterization, especially with new MS technology currently allowing for improved data coverage with low amounts of protein. Through building up the metabolic proteome, we revealed the heterogeneity between leukemia stem/progenitor cells. CD34^+^ displayed high level of TCA and PPP protein expression, supporting a mitochondrial oxidative phosphorylation-dependent phenotype of CML stem cells reported recently. Interestingly, metabolic protein expression of CD34^+^ cells from adult and pediatric AML differ in multiple pathways including TCA and FAO, suggesting differentiated treatment when metabolic targets should be considered (unpublished data). In contrast to the metabolic characteristic of leukemia stem cells described here, normal hematopoietic stem cells were recently shown to display glycolytic advantage including high expression of phosphofructokinase, pyruvate dehydrogenase kinase 2 and 4 [3].

Based on the metabolic proteome, we seek compounds that may reverse the metabolic aberrations. The inhibitory effect of metformin on a number of cancer types as well as its synergistic effect with clinical available compounds have been proposed [47–49]. We expected that metformin could inhibit the increased expression of TCA proteins as well as known high TCA activity in CML stem/progenitor cells, thus result in killing of these cells. We indeed observed strong apoptosis-inducing effect of metformin, however, no expression pattern was rewired. The abundance of TCA enzymes was unexpectedly even higher post metformin treatment, excluding the well-known OXPHOS-inhibiting activity of metformin as the responsible mechanism in killing CD34^+^ leukemia cells. Notably, we observed that ER stress, which is normally induced by nutrient deprivation or hypoxia, was activated and partially mediated the effects of metformin on CD34^+^ leukemia cells [27, 39]. Although metformin monotherapy is not potent enough to conquer the development of CML, it enhanced the sensitivity of CML stem cells to imatinib, suggesting that synergistic use of imatinib with metformin, or ER stress inducer may provide an attractive approach to target BCR-ABL-independent mechanism of resistance.

Taken together, the metabolic proteome resource reveals the previously undescribed characteristic of myeloid leukemia stem/progenitor cells, allowing the identification of novel markers and deep understanding of metabolic reprogram underlying leukemogenesis. The proteomic strategy also designated a novel mechanism beyond mitochondrial oxidative phosphorylation inhibition for metformin, which anti-tumor activity has been extensively evaluated.

## Supplementary information

Supplemental material

Checklist

co-authors’ email responses

## Data Availability

The data supporting the finding of this study are available in the supplementary figures.
