# Supplementary material for: Mitochondrial oxidative phosphorylation is dispensable for survival of CD34+ chronic myeloid leukemia stem and progenitor cells
Source: Cell Death Dis. 2022 Apr 20;13(4):384. doi: 10.1038/s41419-022-04842-5 (PMC9021200; doi:10.1038/s41419-022-04842-5)
Supplement: Supplementary file 1 — Supplemental material [file 41419_2022_4842_MOESM1_ESM.docx]

**Mitochondrial oxidative phosphorylation is dispensable for survival of CD34^+^ chronic myeloid leukemia stem and progenitor cells**

Jin-Song Yan^1,2†*^, Meng-Ying Yang^1†^, Xue-Hong Zhang^3†^, Chen-Hui Luo^4†^, Cheng-Kan Du^1^, Yue Jiang^2^, Xuan-Jia Dong^1^, Zhang-Man Wang^2^, Li-Xue Yang^1^, Yi-Dong Li^5*^, Li Xia^6*^, Ying Lu^1,2*^

**Supplemental Figures**

**Supplemental Figure 1. Hierarchical clustering analysis of CD34^+^ between CML and normal samples.** Clustering was done using Euclidean distance upon differential expression genes obtained by the limma package (FC > 2 and *P* < 0.01). Quantile normalization was applied prior to the analysis.


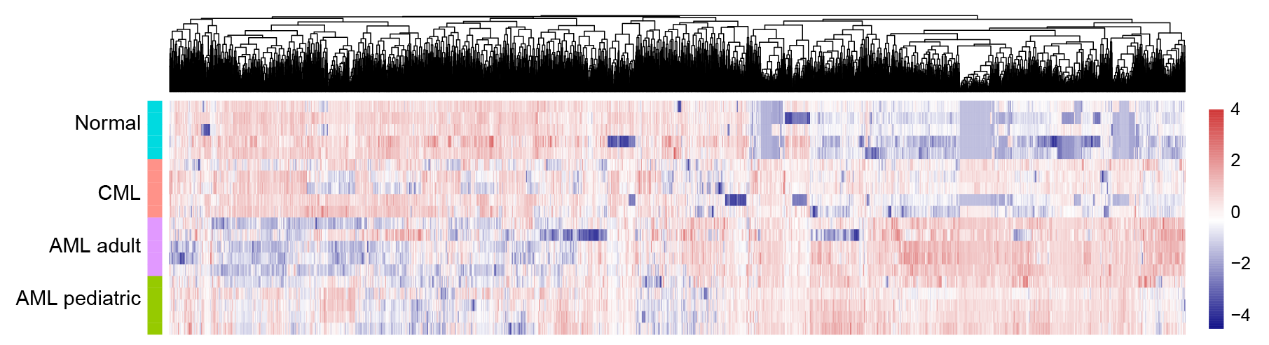

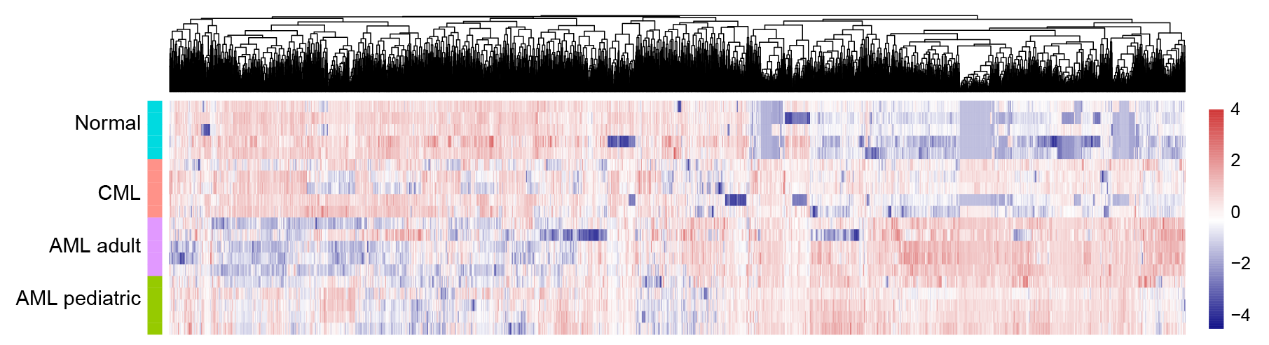


**Supplemental Figure 2. Ingenuity Pathway Analysis (IPA) analysis of central carbonate metabolic pathways.** The up-regulated proteins are colored in pink, and the down-regulated genes are colored in green.

**
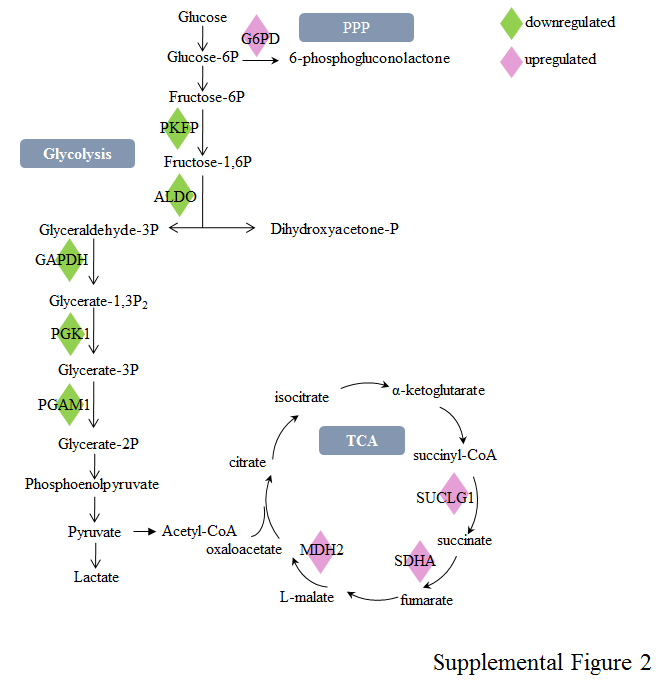
**

**Supplemental Figure 3. Metformin induced cell cycle arrest of K562 cells.** (A) K562 cells were treated with 0.5 µM imatinib, 8mM metformin or combination of imatinib with metformin for 72 hours. Cell apoptosis were measured by AnnexinV/PI staining. Representative flow cytometry plots were shown with percentage of cell death. (B) Cell-cycle distribution, as measured by PI staining of K562 cells treated with (right) or without (left) metformin for 48 h. Data are representative of three independent experiments.

**
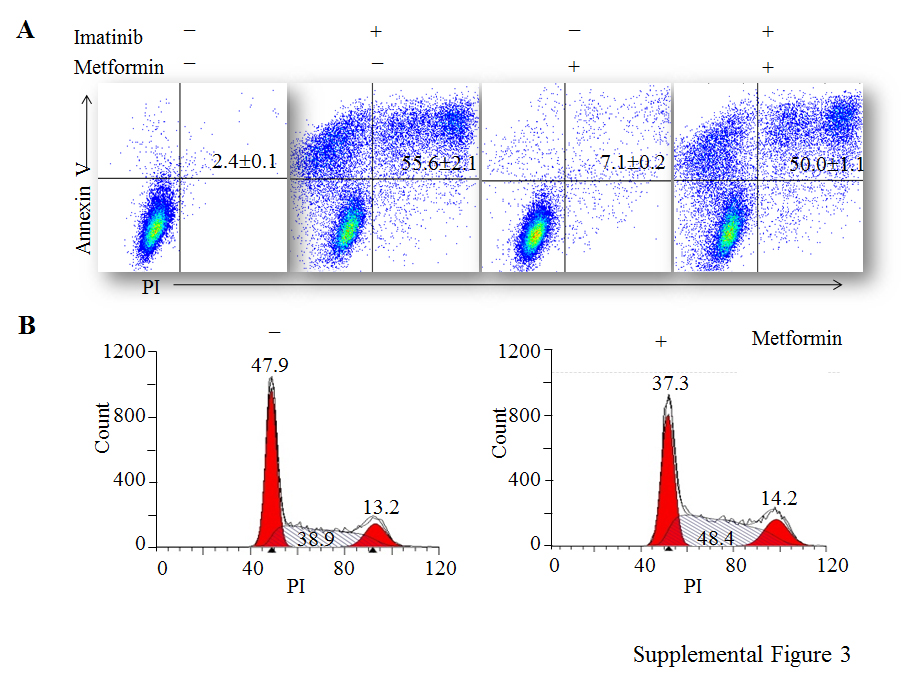
Supplemental Figure 4. ER stress inhibitor MKC8866 partially blocked the apoptosis-inducing effect of metformin.** Purified CML CD34^+^ cells were treated with metformin, MKC8866 or MKC8866 plus metformin for 72 hours. Cell apoptosis were measured. Representative flow cytometry plots were shown on the top. *n* = 3.


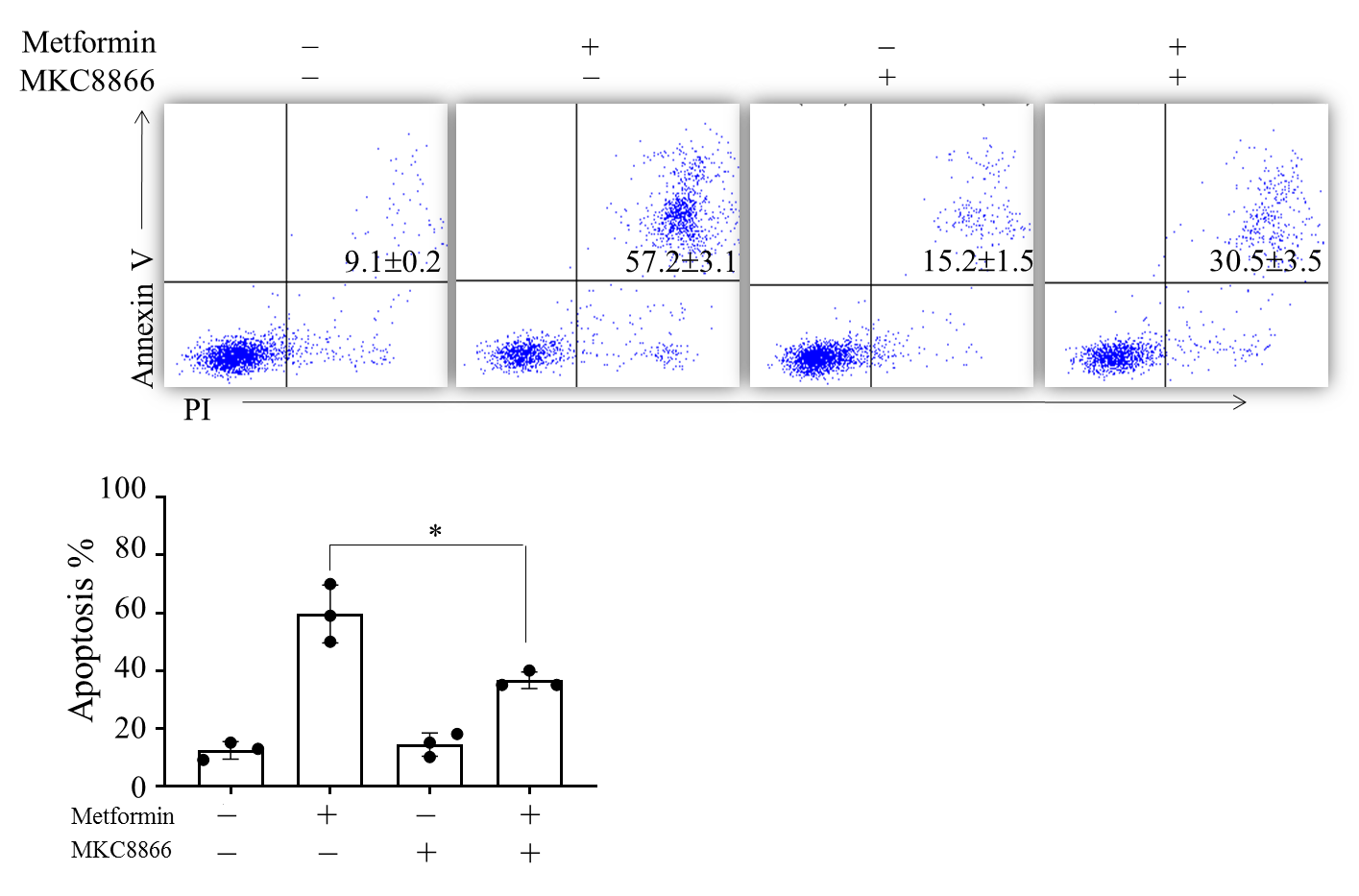


**Supplemental Figure 5. Unedited gels for all the western blot bands in the manuscript.**

**
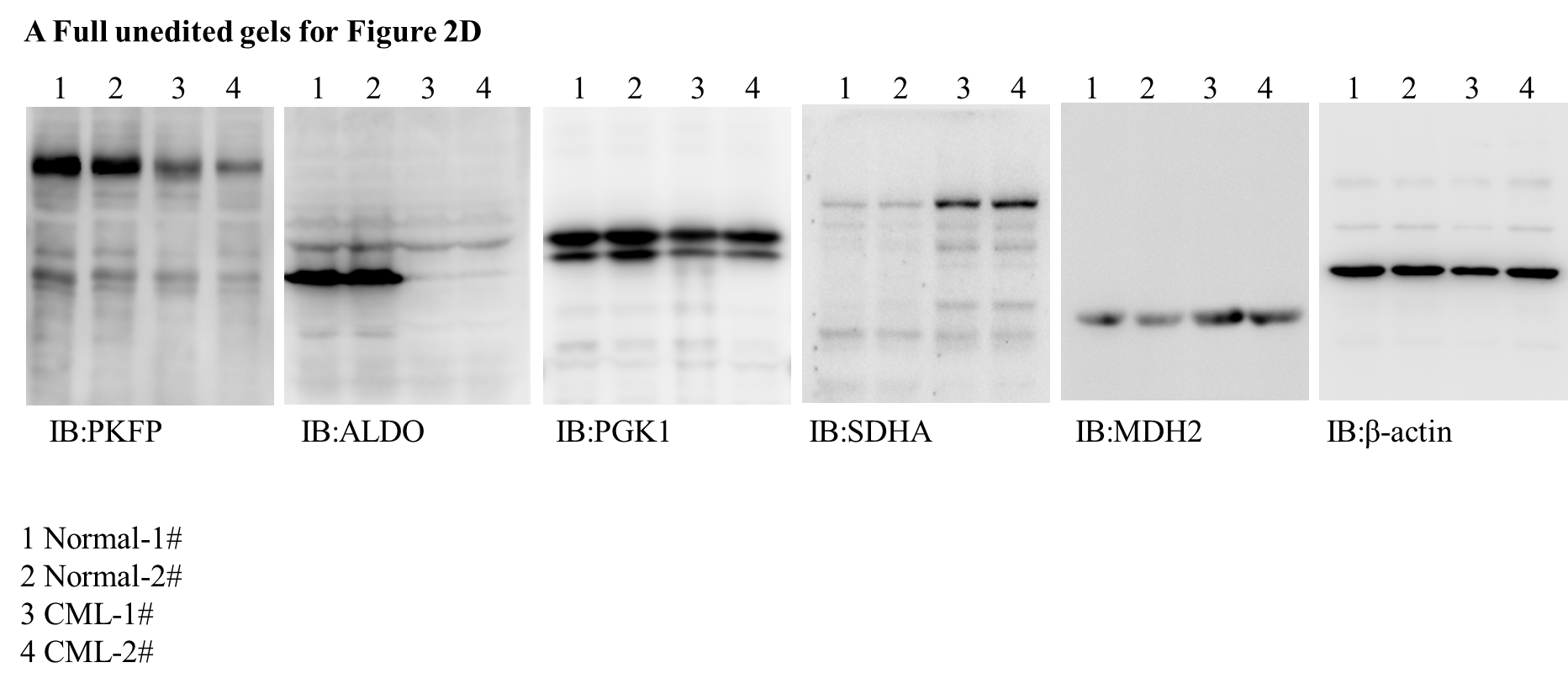
**

**
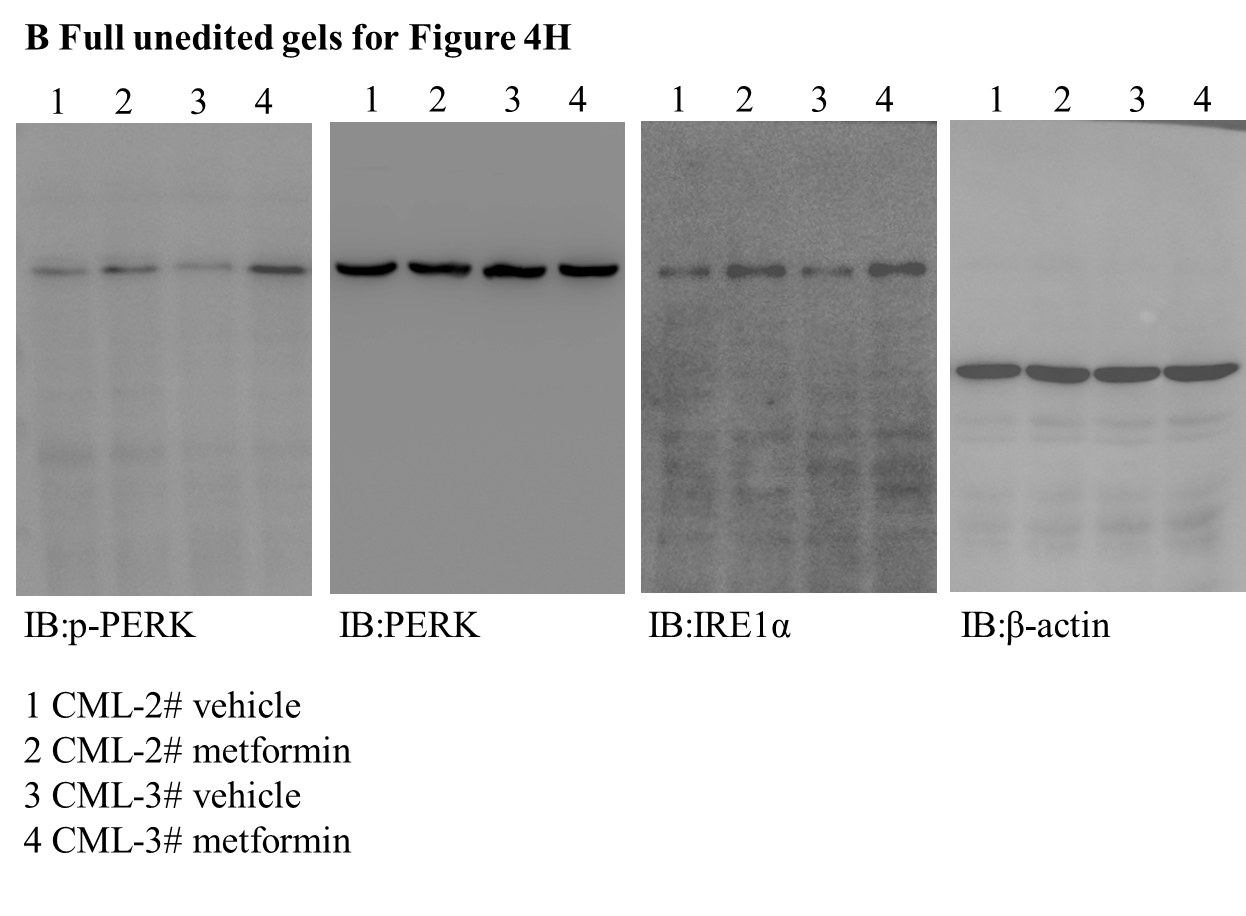
**

**Supplemental Methods**

**Targeted analysis on glucose metabolism**

The metabolites were detected by using HILIC-UPLC-QTOF-MS. The HILIC-UPLC-QTOF-MS system was equipped with a LC30AD^TM^ UPLC (Shimadzu, Kyoto, Japan) coupled with a TripleTOF^TM^ 5600 plus (Sciex, MA, USA) QTOF mass spectrometer under negative ion mode. A Waters ACQUITY^®^ UPLC BEH Amide column (1.7 μm, 100 mm × 2.1 mm) was used for chromatographic separation. The column temperature was set at 45 °C, flow rate 0.40 mL/min, sample injection volume 5 μl. The metabolites were separated over a linear gradient from 5% B to 70% B for 10min (mobile phase A (95% ACN, 0.1% formic acid, 10mM ammonium formate) and B (95% H_2_O, 0.1% formic acid, 10mM ammonium formate)). The peak areas of the different metabolites were calculated and determined using Sciex Multiquant software through which the exact mass of the singly charged ion and retention time was analyzed by the commercially available standard compounds using the same HILIC-UPLC-QTOF-MS system.
