# Supplementary figures and images for: Mitochondrial oxidative phosphorylation is dispensable for survival of CD34+ chronic myeloid leukemia stem and progenitor cells
Source: Cell Death Dis. 2022 Apr 20;13(4):384. doi: 10.1038/s41419-022-04842-5 (PMC9021200; doi:10.1038/s41419-022-04842-5)

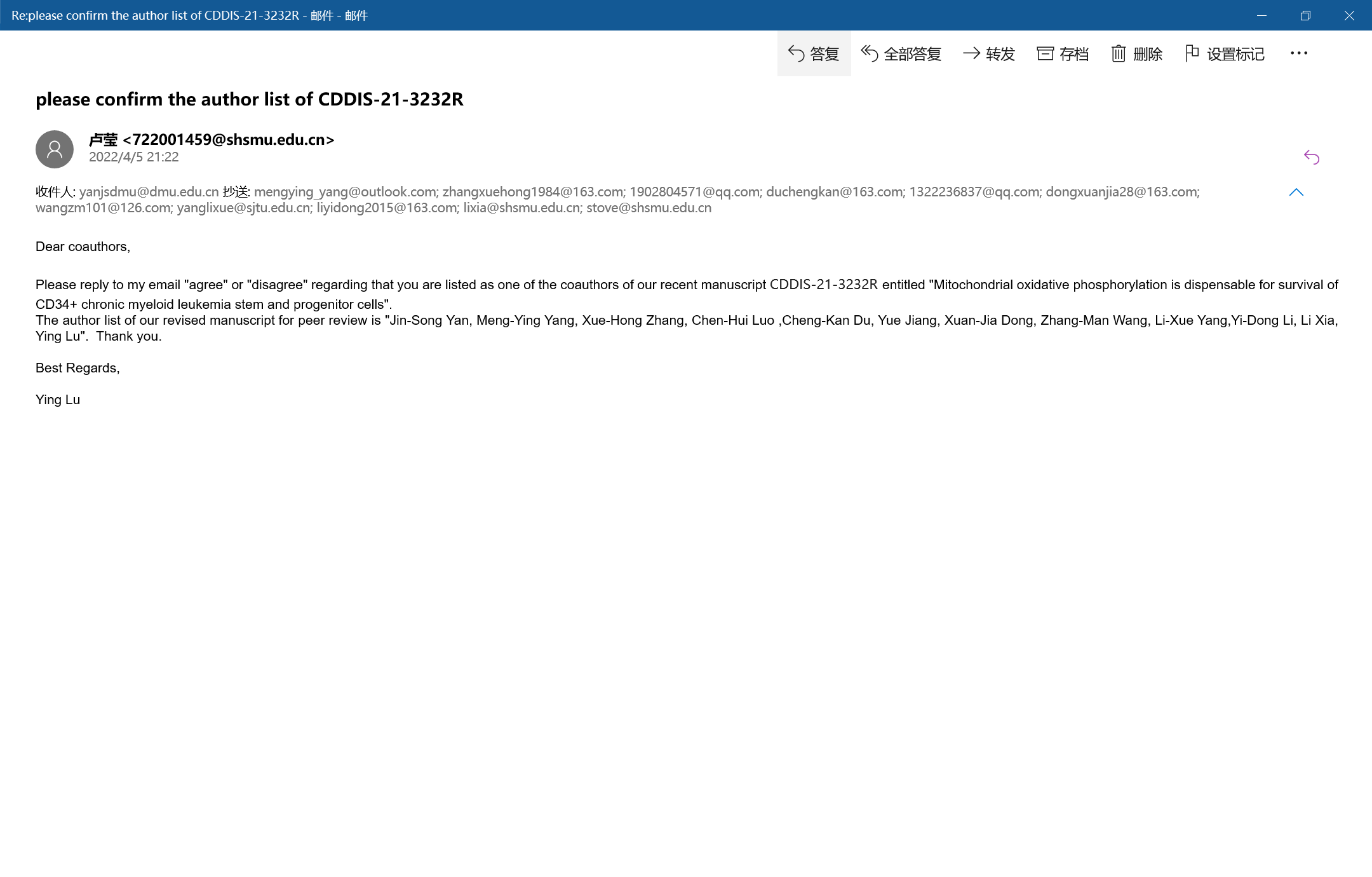


Co-authors’ email responses:


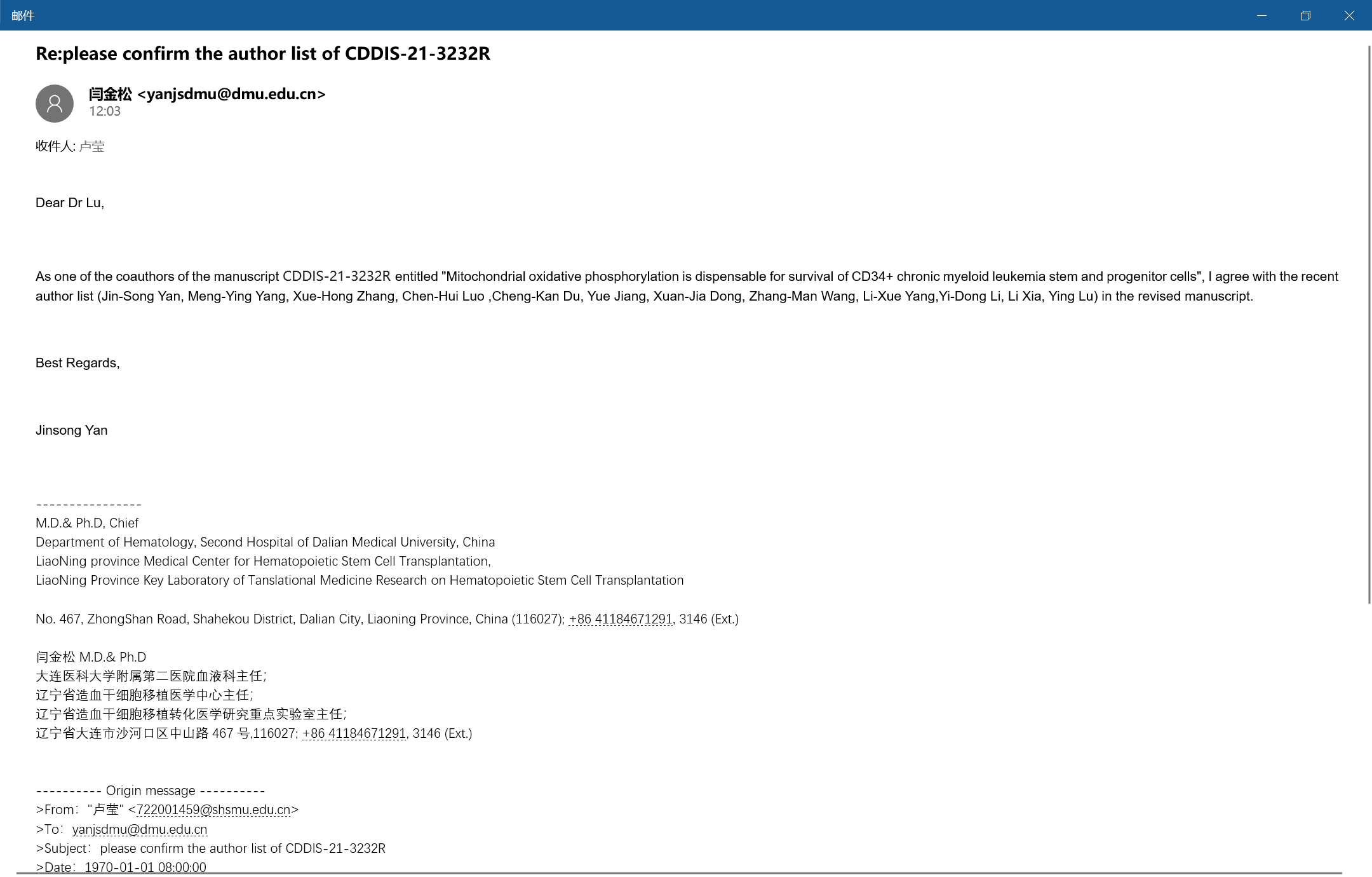


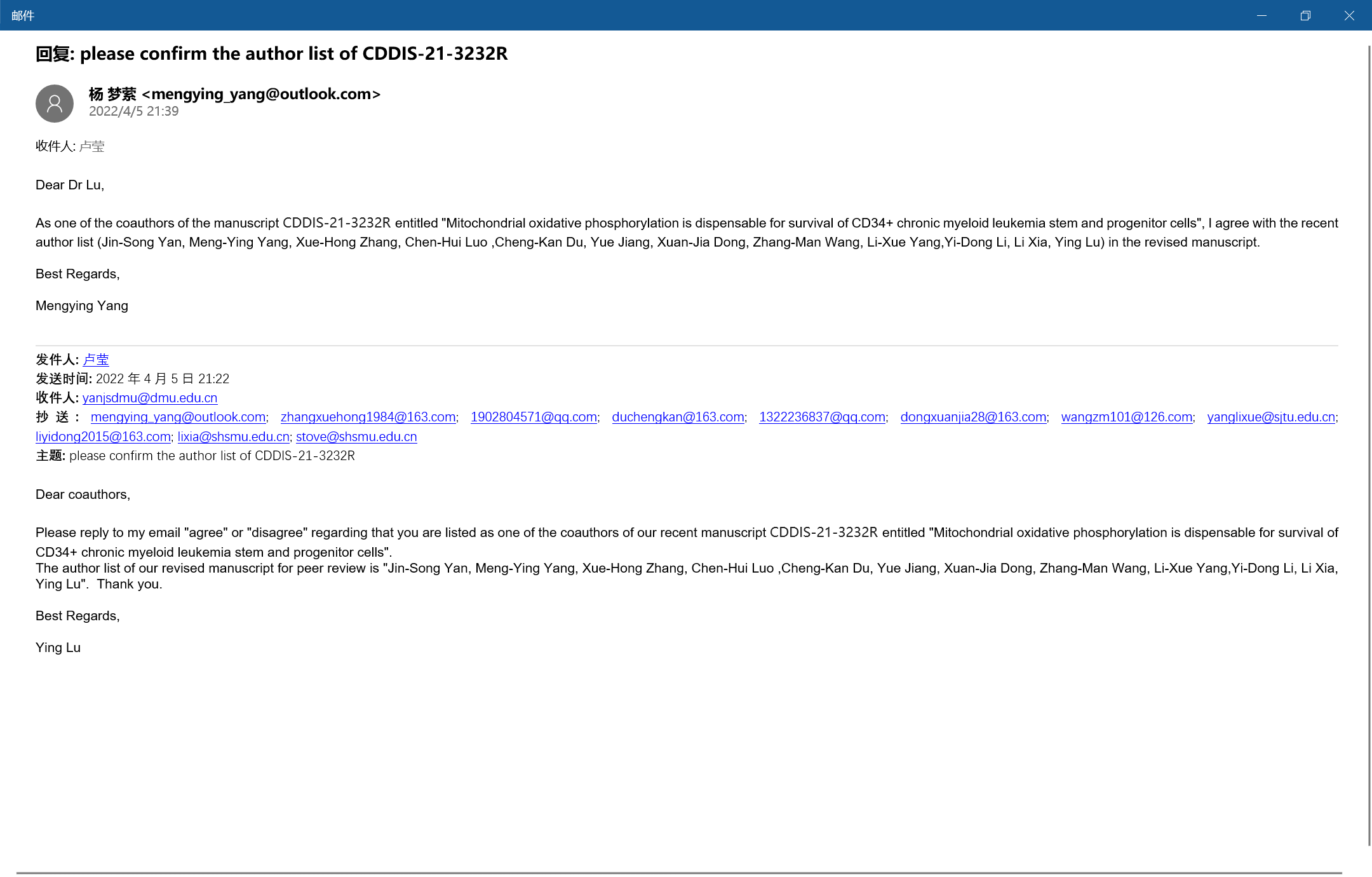


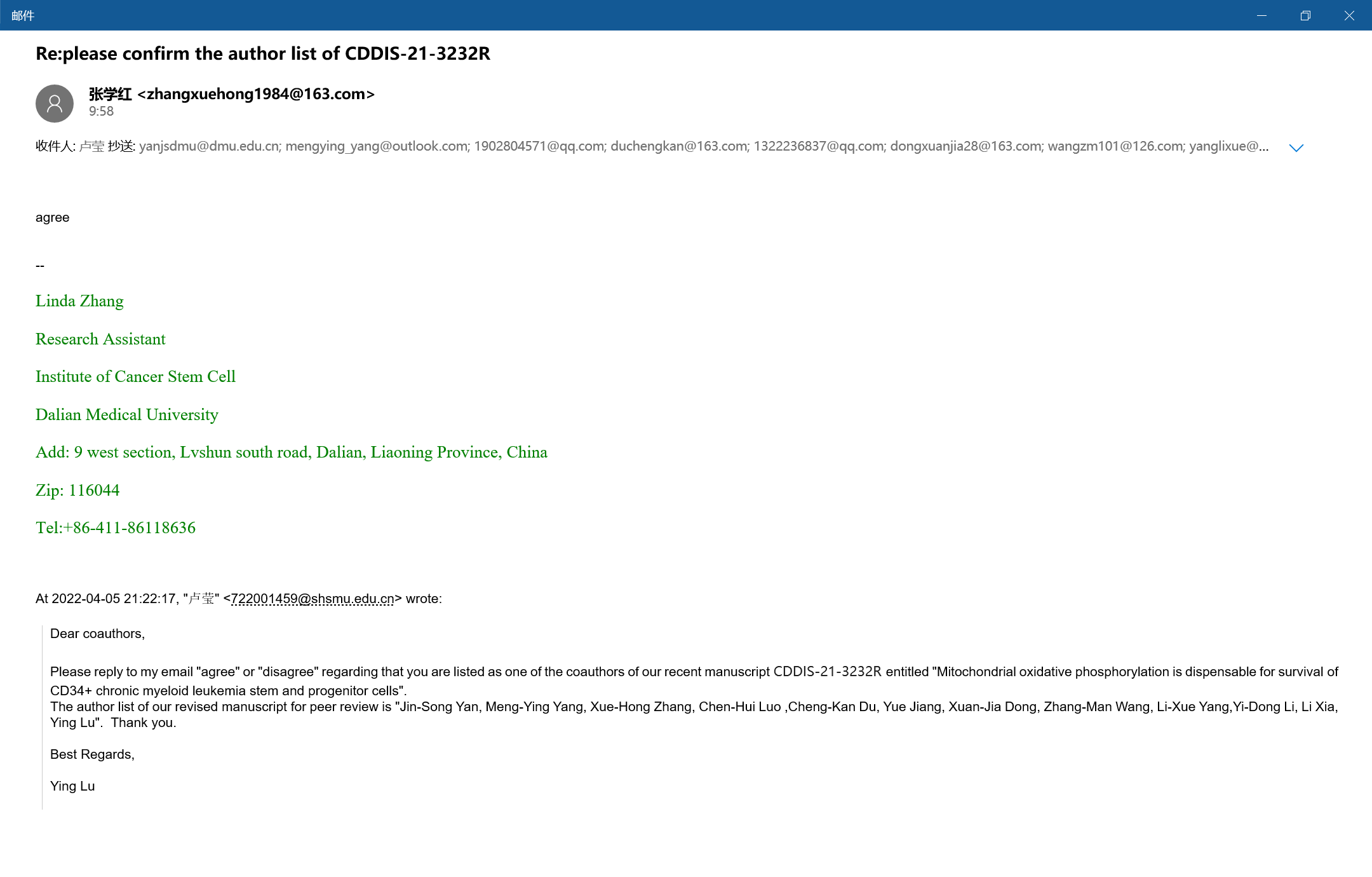


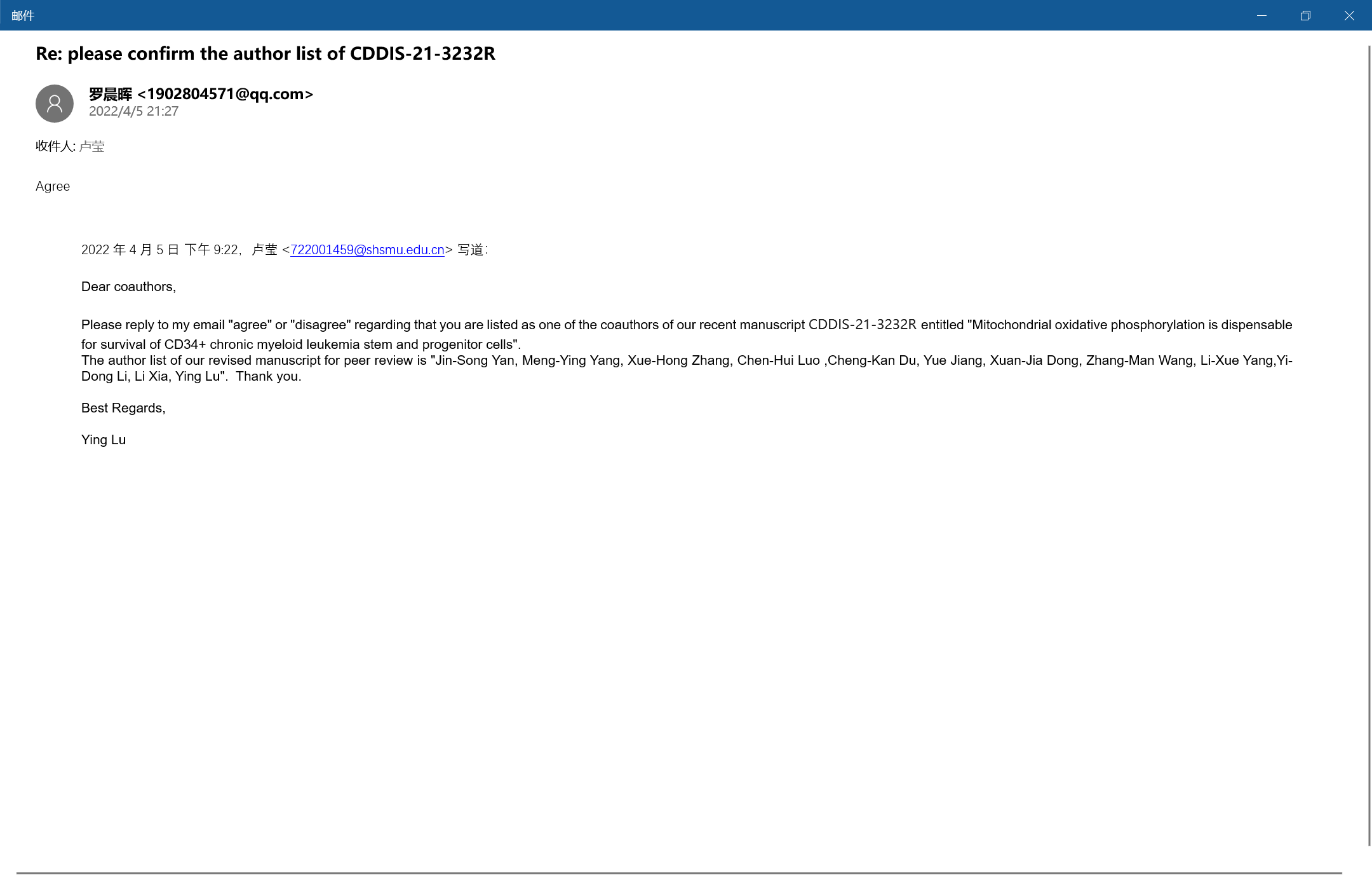


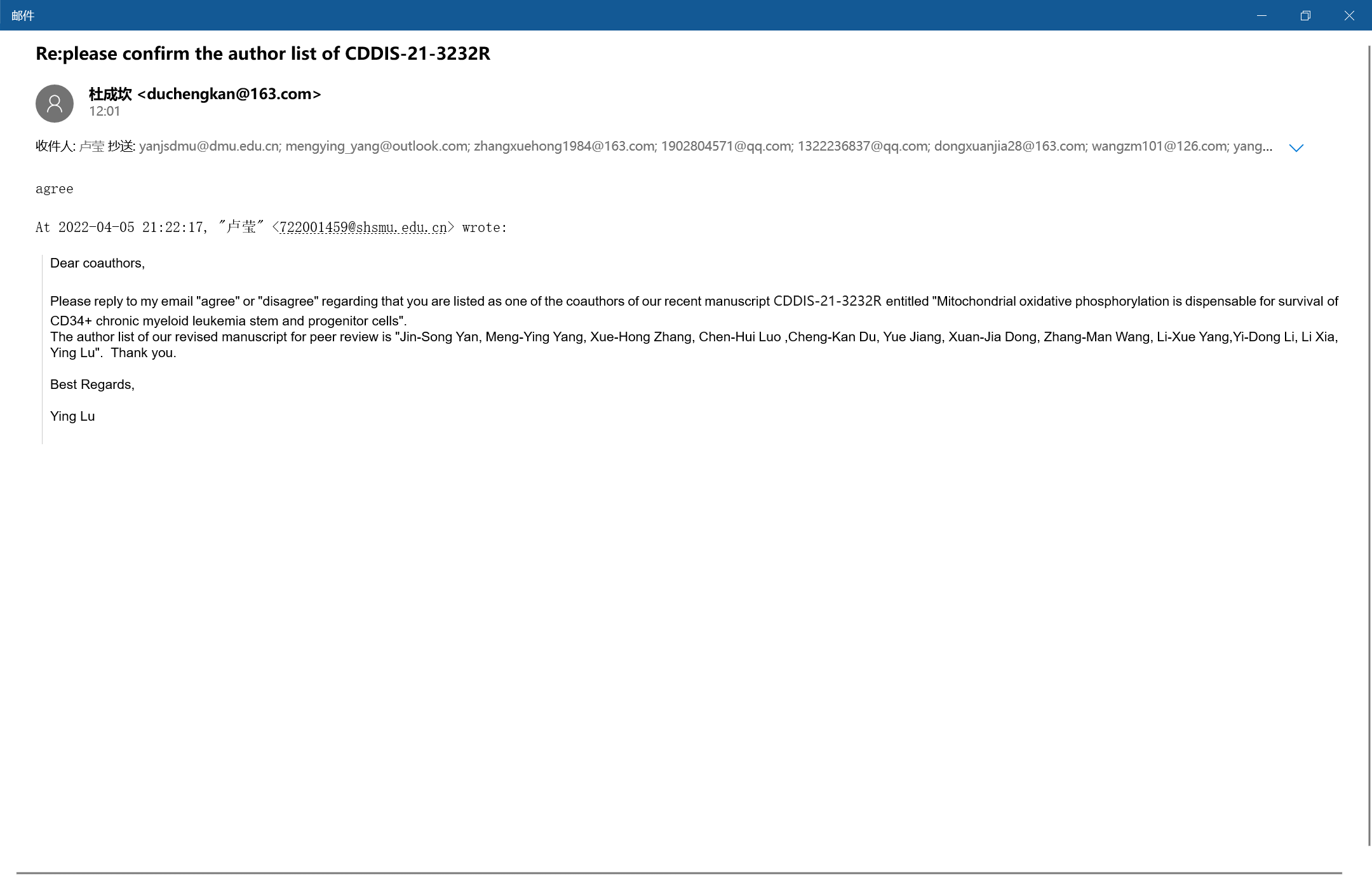


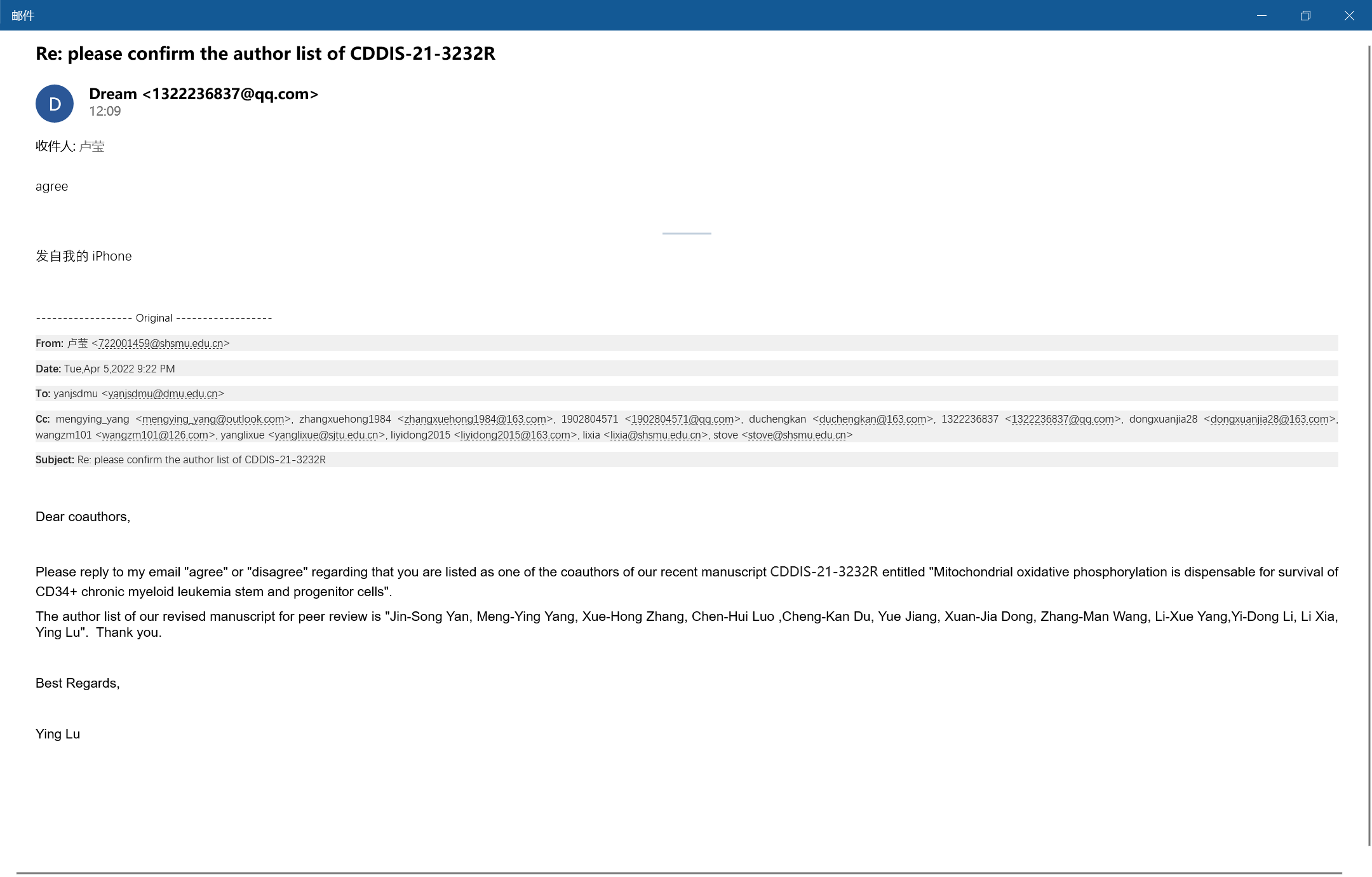


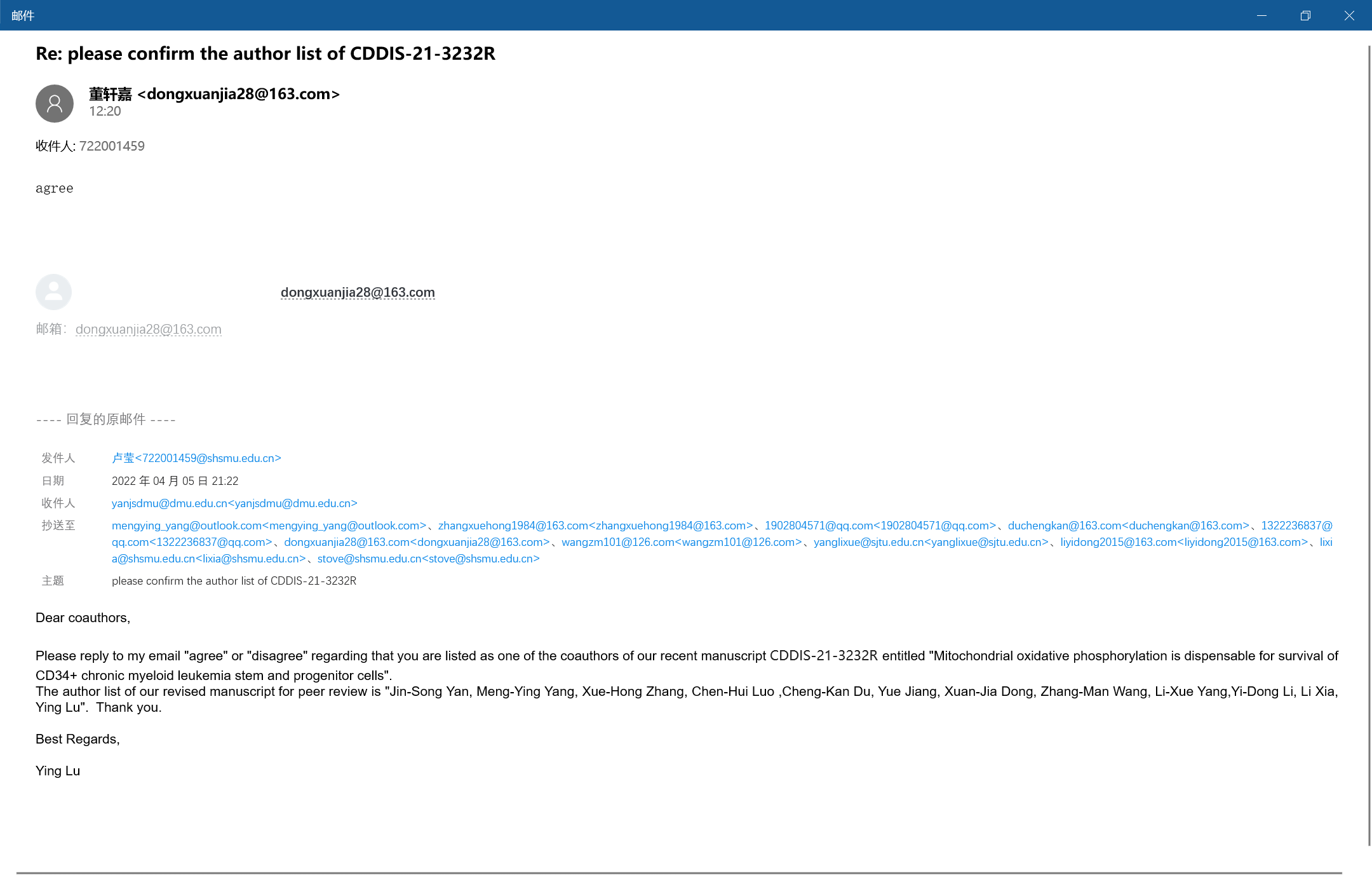


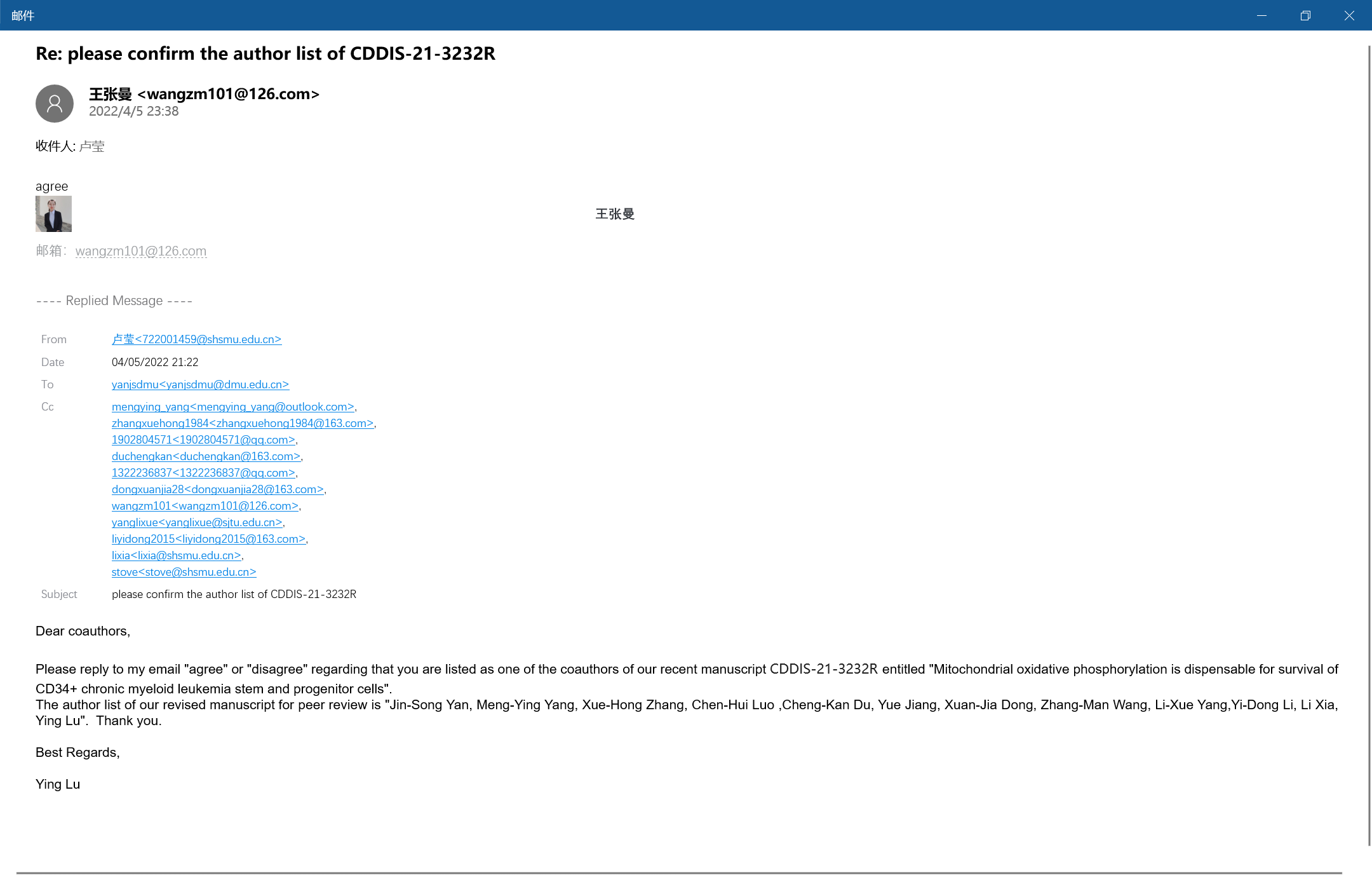


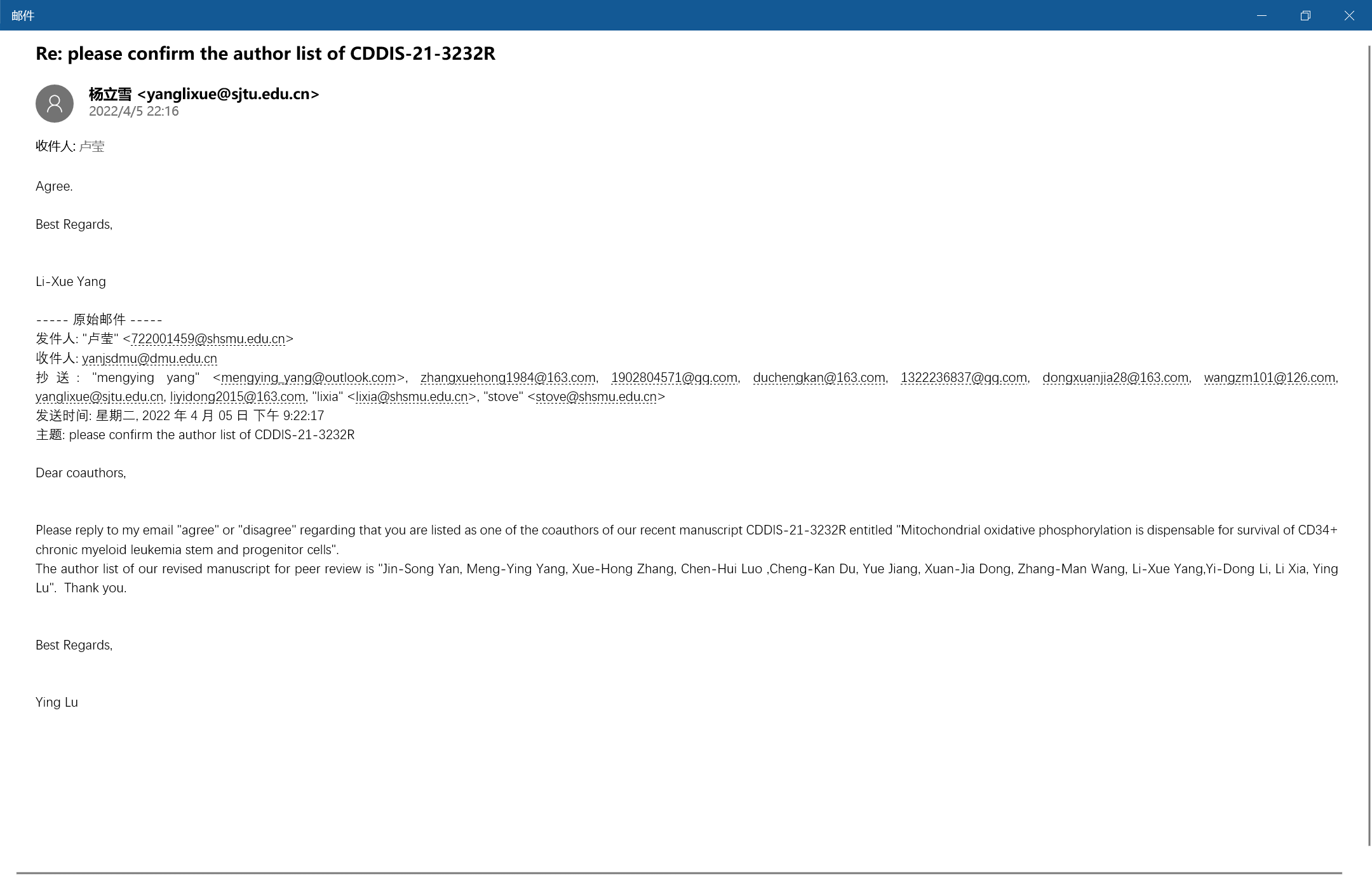


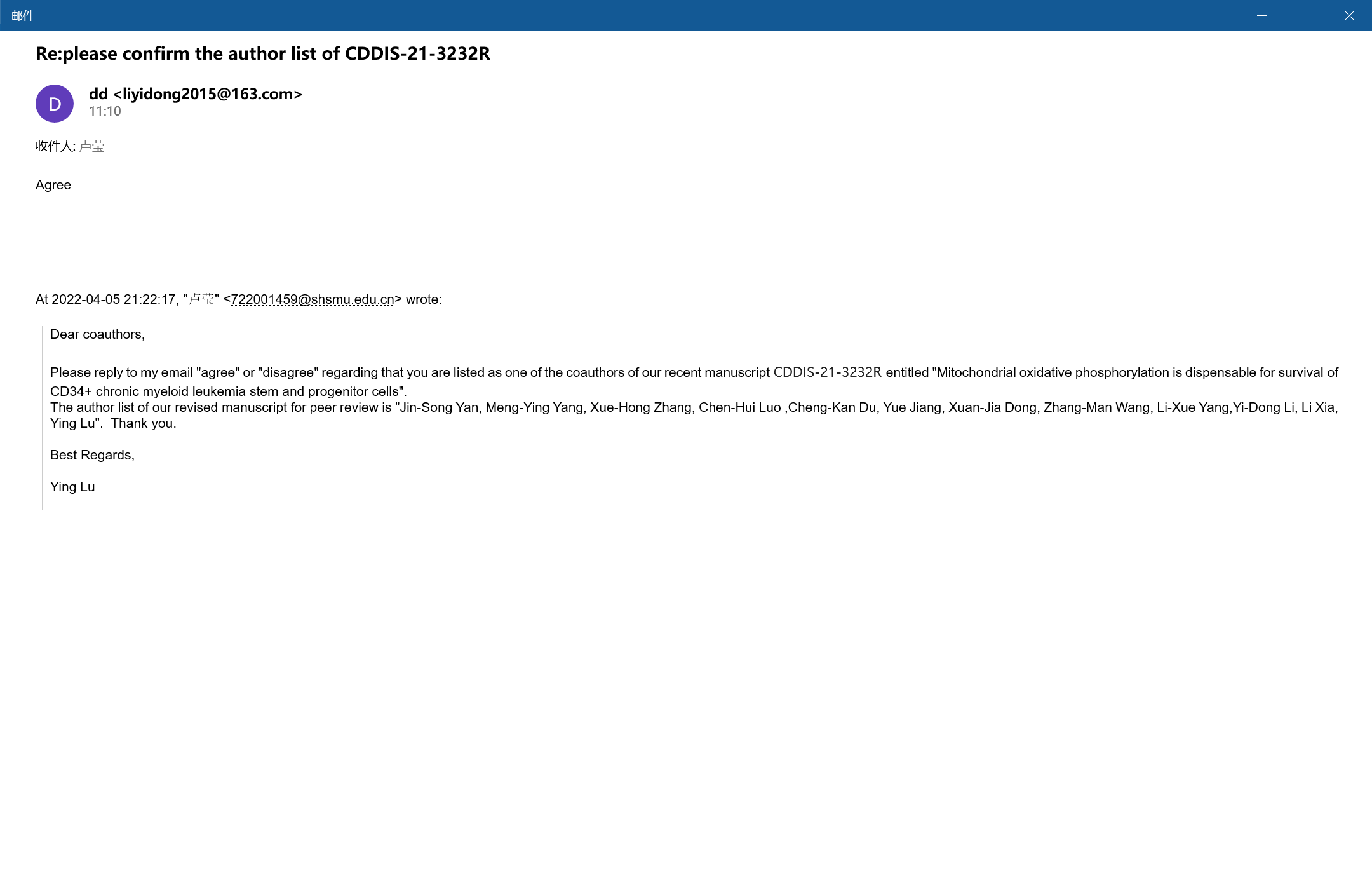


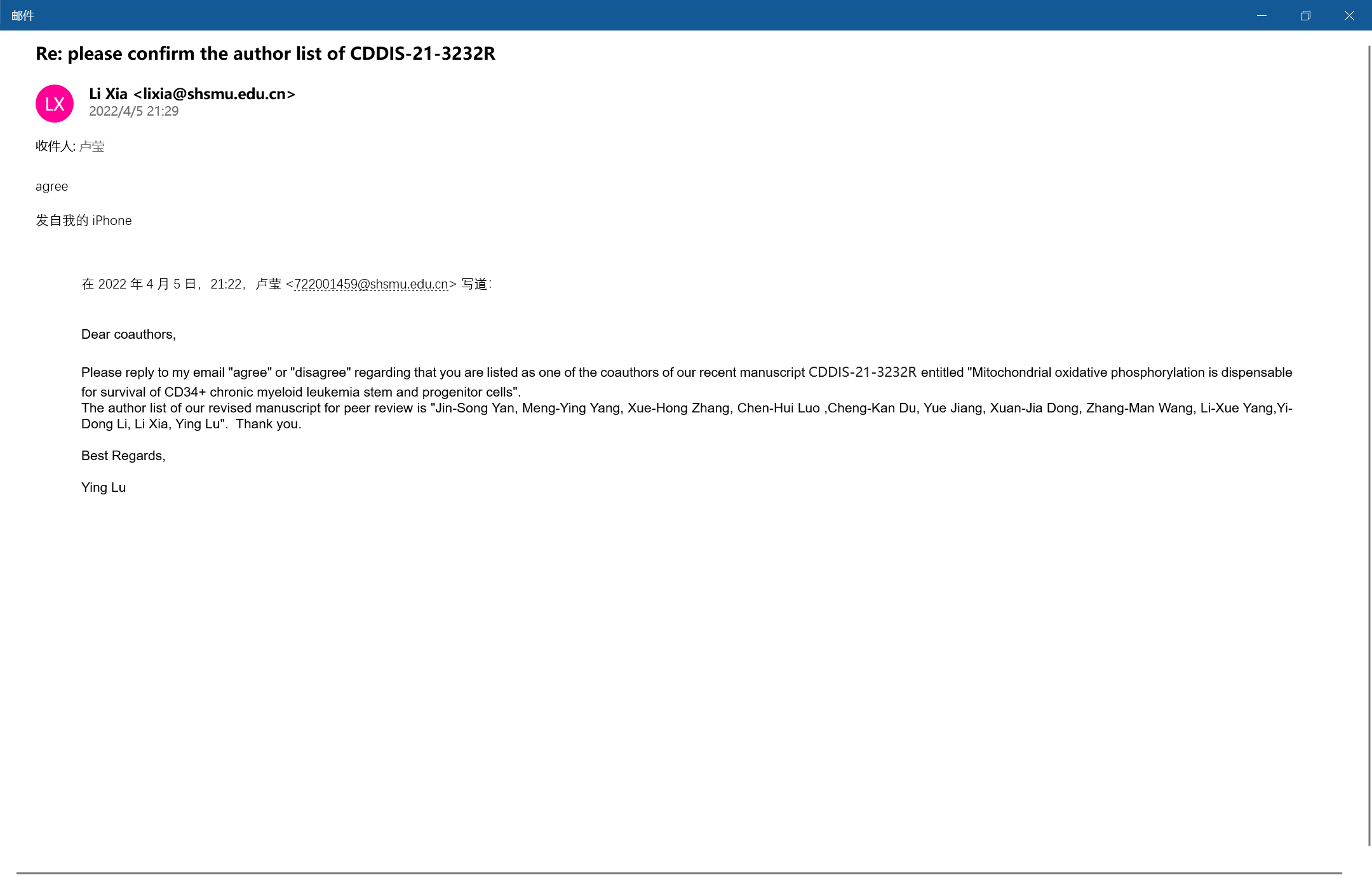

Supplement: Supplementary file 3 — co-authors’ email responses [file 41419_2022_4842_MOESM3_ESM.docx]
